# Comparative Diagnostic Performance of Conventional and Novel Fatty Acid Indices in Blood Plasma as Biomarkers of Atherosclerosis Under Statin Therapy

**DOI:** 10.3390/biomedicines14010149

**Published:** 2026-01-11

**Authors:** Nikolay Eroshchenko, Elena Danilova, Anastasiia Lomonosova, Philipp Kopylov, Svetlana Lebedeva, Andreas Tsakalof, Alexander Nosyrev

**Affiliations:** 1 Institute of Molecular Theranostics, Biomedical Science and Technology Park, Sechenov University, 119048 Moscow, Russia; phenolyat@gmail.com (E.D.); nosyrev_a_e@staff.sechenov.ru (A.N.); 2 Institute for Personalized Cardiology, Biomedical Science and Technology Park, Sechenov University, 119048 Moscow, Russia; lomonosova_a_a@staff.sechenov.ru (A.L.); kopylov_f_yu@staff.sechenov.ru (P.K.); 3 Rehabilitation Room No. 2, University Clinical Hospital No. 1, Sechenov University, 119048 Moscow, Russia; 4 Department of Cardiology, Functional and Ultrasound Diagnostics, N.V. Sklifosovsky Institute for Clinical Medicine, Sechenov University, 119048 Moscow, Russia; 5 Department of Pharmacology, Institute of Pharmacy Named After A.P. Nelyubin, Sechenov University, 119048 Moscow, Russia; lebedeva502@yandex.ru; 6 Department of Medical Elementology, Peoples’ Friendship University of Russia (RUDN University), 117198 Moscow, Russia; 7 Laboratory of Biochemistry, School of Medicine, University of Thessaly, Biopolis, 41111 Larissa, Greece

**Keywords:** atherosclerosis, statin therapy, lipidomics, fatty acid metabolism, omega-3, omega-6, EPA, DHA, adrenic acid, biomarker

## Abstract

**Background:** Atherosclerosis and its associated chronic inflammation of the arterial wall disrupt fatty acid metabolism, leading to changes in plasma fatty acid composition. These alterations can be used to improve disease diagnosis and risk stratification by the development and application of specific lipidomic indices. **Objectives:** The objectives of this study are to evaluate the performance of conventional fatty acid indices and enhance diagnostic efficiency in atherosclerosis by introducing novel index based on plasma PUFA n-6 and n-3 content (Omega-6/3 Balance Index, O6/3-BI), as well as the perspective SFA/MUFA ratio (stearic/oleic acid ratio, C18:0/C18:1n-9) and a logit function combining PUFA and SFA/MUFA biomarkers. **Methods:** Plasma fatty acids were quantified by LC-MS/MS in healthy controls (*n* = 50) and patients with carotid atherosclerosis (*n* = 52), stratified by atorvastatin, rosuvastatin, or no statin therapy. The conventional indices (the Omega-3 Status (EPA + DHA), AA/EPA, and the omega-6/omega-3 ratio), and pathway ratios (C18:0/C18:1n-9; and C20:4n-6/C22:4n-6), as well as the newly introduced PUFA index and combined PUFA-SFA/MUFA logit function, were calculated. Their diagnostic performance for distinguishing atherosclerosis was assessed by a receiver operating characteristic (ROC) analysis with the cross-validation and calculation of Cliff’s Δ effect size. **Results:** The conventional parameters demonstrated a poor to low discrimination ability of the atherosclerosis patients’ groups from healthy controls (area under the ROC curve, AUC 0.548–0.711). In statin-treated patients, these conventional markers lost significance. The newly introduced PUFA index and SFA/MUFA ratio demonstrated improved patients’ discrimination with AUC 0.734–0.780 for the former and strong predictive power with AUC 0.831–0.858 for the latter marker and maintained their diagnostic value under statin therapy. The most significant positive effect size was observed for the SFA/MUFA ratio with Cliff’s Δ = 0.67–0.71. The combined PUFA-SFA/MUFA logit function also demonstrated a strong predictive power with AUC = 0.880 (Cliff’s Δ = −0.76), outperforming any single index. **Conclusions:** The newly introduced lipidomic index based on the PUFA content, SFA/MUFA ratio, and a logit function combining PUFA-SFA/MUFA biomarkers demonstrated a substantially better discrimination of atherosclerosis-related fatty acid metabolic disturbances than conventional fatty acid biomarkers.

## 1. Introduction

Atherosclerosis is the leading cause of cardiovascular disease and mortality worldwide [1]. Population-level screening for subclinical (“silent”) atherosclerosis and initiating treatment before it becomes life-threatening are considered among the key goals of modern cardiovascular prevention programs [2,3]. The monitoring of biological compounds involved in the development of atherosclerosis or metabolically influenced by associated pathophysiological processes can provide valuable predictive biomarkers for the screening and early detection of atherosclerosis or the evaluation of atherosclerosis development risk [4].

Advances in understanding the molecular mechanisms of atherosclerosis have underscored the central role of lipid metabolism in sustaining vascular inflammation and plaque instability. This has given rise to the search for novel diagnostic approaches that can help to monitor therapeutic responses effectively [5,6]. Among lipid-related biomarkers, fatty acids have gained significant attention because they directly influence inflammation, endothelial function, and lipid metabolism, which are key processes in arterial disease development [7]. Fatty acid composition can be measured in plasma, serum, or red blood cell (RBC) membranes, which allows us to evaluate both recent dietary intake and long-term fatty acid status [8].

Multiple studies have demonstrated that specific fatty acid-derived indices that are calculated from ratios or combinations of individual fatty acids serve as powerful predictors of cardiovascular events and atherosclerotic progression [9]. Their diagnostic value lies in their ability to reflect the interplay between dietary habits, metabolism, inflammation, and vascular health. Long-term prospective investigations have validated the clinical relevance of fatty acid profiling in cardiovascular risk prediction [10]. Within this context, polyunsaturated fatty acids (PUFAs) including omega-3 and omega-6 species have attracted considerable attention. They function both as essential nutrients and as modulators of inflammatory processes [11].

Omega-3 fatty acids such as eicosapentaenoic acid (EPA) and docosahexaenoic acid (DHA) influence lipid profiles and serve as precursors to specialized pro-resolving mediators (SPMs) that promote the resolution of inflammation [12,13]. Through mechanisms that include the attenuation of leukocyte infiltration and cytokine production, SPMs contribute to vascular homeostasis and plaque stabilization [14,15].

Several established indices have emerged as clinically relevant biomarkers, including the Omega-3 Status (EPA + DHA), the AA/EPA ratio, and various omega-6 to omega-3 ratios [9,16]. Omega-3 Status can be assessed using the “Omega-3 Index”, defined as the proportion of EPA and DHA in RBC membranes, which has been associated with cardiovascular protection. Values of 8 percent or higher indicate the lowest cardiovascular risk, whereas values of 4 percent or lower identify individuals at an increased risk [17,18,19,20]. The arachidonic acid (AA)-to-EPA ratio (AA/EPA) has also been validated as a marker of atherosclerotic risk, because higher AA/EPA ratios reflect a shift toward a more pro-inflammatory state [21,22,23]. The ratios of omega-6 to omega-3 fatty acids have likewise been widely studied, although their clinical utility remains debated [17,24]. Among large clinical trials, the Japan EPA Lipid Intervention Study (JELIS) demonstrated that EPA supplementation in statin-treated patients reduced major coronary events by 19 percent and identified the AA/EPA ratio as an independent predictor of future cardiovascular events [25].

Although PUFAs have historically been the primary focus of fatty-acid-based cardiovascular biomarkers, interest has expanded toward monounsaturated fatty acids (MUFAs), particularly oleic acid (C18:1n-9) [26]. MUFAs are major components of dietary fats and are synthesized endogenously from saturated fatty acids (SFAs) by stearoyl-CoA desaturase-1 (SCD1) [27]. Alterations in the activity of this enzyme, which can be reflected by the stearic-to-oleic acid ratio (C18:0 to C18:1n-9), are associated with metabolic health, insulin sensitivity, and changes in the tissue lipid content [28,29,30]. Epidemiological data indicate that a higher saturated fatty acid intake is associated with increased coronary heart disease mortality, while MUFA intake shows an inverse association [10]. These findings support the potential value of the SFA-to-MUFA ratio as a dietary and metabolic biomarker [31]. Nevertheless, studies evaluating MUFAs as cardiovascular biomarkers have produced inconsistent results, which highlights the need for further investigation [26,32].

Despite the progress in the diagnostics and treatment of atherosclerosis, many patients with low circulating omega-3 levels continue to show low-grade vascular inflammation (residual inflammatory risk) and plaque progression even under effective statin therapy and low-density lipoprotein cholesterol (LDL-C) control [5,33,34]. This observation has prompted interest in more refined lipidomic indices that capture PUFA metabolic pathway dynamics more comprehensively [35]. As a result, clinical laboratories increasingly measure not only the absolute levels of PUFAs but also their relative ratios, which include the Omega-3 Status (EPA+DHA), AA/EPA ratio, and omega-6-to-omega-3 ratio [9,16].

New biomarkers continue to emerge. Adrenic acid (AdA), which is generated through a two-carbon elongation of arachidonic acid by elongase enzymes, has attracted interest because of its anti-inflammatory properties [36]. Changes in the AA-to-AdA ratio can shift the balance of inflammatory mediator production, and AdA supplementation has demonstrated beneficial effects in experimental inflammatory models [37]. However, the relevance of this ratio in atherosclerosis remains insufficiently investigated.

The integration of multiple fatty acid patterns and desaturase indices may offer a more comprehensive assessment of cardiovascular risk compared to the evaluation of individual markers. Patients with cardiovascular disease exhibit characteristic alterations in fatty acid composition and desaturase activity, and combined indices may capture the metabolic shifts associated with atherosclerosis more effectively [38,39]. Nevertheless, no biomarker currently integrates several enzymatic endpoints of PUFA metabolism into a single diagnostic tool [9].

Our previous work demonstrated that patients with carotid atherosclerosis exhibit significant changes in plasma and plaque fatty acid profiles, including increased MUFAs and reduced omega-3 PUFAs, which suggest alterations in elongase and desaturase activities [40]. These metabolic shifts were associated with unstable plaque phenotypes and reduced levels of SPM precursors [40]. These findings support the development of more comprehensive indices that can detect fatty acid metabolic disruptions in atherosclerosis more effectively.

In the present study, we propose and validate a novel plasma fatty acid biomarker, the Omega-6-to-Omega-3 Balance Index (O6/3-BI), derived from n-6 and n-3 PUFAs. We also evaluate the diagnostic potential of the stearic-to-oleic-acid ratio (C18:0 to C18:1n-9), which represents the SFA-to-MUFA balance, as well as a combined logit function that integrates PUFA and SFA to MUFA indices. We compare the diagnostic performance of conventional fatty acid indices, including the Omega-3 Status (EPA + DHA), AA/EPA, and omega-6/omega-3 ratios, with these new metrics for the detection of fatty acid alterations associated with atherosclerosis. In addition, we assess the effect of statin therapy on the fatty acid composition and on the values of both established and novel indices, because the influence of statins on the PUFA metabolism and specialized pro-resolving mediator pathways has not been thoroughly studied [6,22,41].

## 2. Materials and Methods

### 2.1. Study Design and Participants

This study represents a direct continuation of our previously published work on fatty acid profiling in patients with carotid atherosclerosis. The full design of the case–control study, including ethics approval (Sechenov University Ethics Committee, Protocol Code No. 16-22, 1 September 2022), patient recruitment criteria, sample collection, analytical procedures, and statistical approach, has been described in detail in the original publication [40]. The patients provided written informed consent. No changes were made to the study design, inclusion criteria, sample preparation, or analytical techniques. The detailed inclusion and exclusion criteria are presented in Table S1. 

Briefly, the study included 52 patients undergoing carotid endarterectomy at Sechenov University, forming atherosclerosis group (AS). Plasma samples were collected following an overnight fasting and analyzed using previously developed and validated liquid chromatography–tandem mass spectrometry (LC-MS/MS) method to determine their fatty acid composition [42]. A brief overview of the sample preparation and HPLC-MS/MS conditions can be found in Table S2.

Comparative analyses were performed using a control group (Control) of 50 apparently healthy individuals, free of clinical or imaging signs of atherosclerosis, who were previously recruited and analyzed using identical plasma sampling and LC-MS/MS protocols. This control group serves as a baseline for assessing deviations in fatty acid profiles in the atherosclerosis subgroups.

List of analyzed fatty acids: saturated (C12:0, C14:0, C16:0, C17:0, C18:0, C20:0, C22:0, and C24:0) and unsaturated omega-7 (C16:1n-7), omega-9 (C18:1n-9, C20:1n-9, and C24:1n-9), trans fatty acids (C16:1n-7 *trans*, C18:1n-9 *trans*, and C18:2n-6 *trans*), omega-6 (C18:2n-6, C18:3n-6, C20:2n-6, C20:3n-6, C20:4n-6 and C22:4n-6), and omega-3 (EPA C20:5n-3, DPA C22:5n-3, and DHA C22:6n-3).

The percentage content of each fatty acid was calculated as molar percentage. First, each fatty acid measured by LC-MS/MS (ng/mL) was converted to a molar amount by dividing by its molecular weight. Then, for each sample, we summed the molar amounts of all fatty acids quantified in our panel and expressed each fatty acid as its molar amount divided by this total × 100. Fatty acids below the lower limit of quantification were not included in the total.

The Omega-3 Status (EPA + DHA) was calculated as the sum of EPA and DHA, expressed as a molar percentage of the total quantified plasma fatty acids. The omega-6/omega-3 ratio was calculated as the sum of n-6 PUFAs (C18:2n-6, C18:3n-6, C20:2n-6, C20:3n-6, C20:4n-6 and C22:4n-6) divided by the sum of n-3 PUFAs (EPA, DPA and DHA).

### 2.2. Stratification by Statin Therapy

Patients from AS group (*n* = 52) were retrospectively stratified into three subgroups based on their statin treatment status at the time of sample collection. The groups included 21 patients receiving rosuvastatin, 19 receiving atorvastatin, and 12 without statin therapy. Statin therapy in all statin-treated groups lasted at least 6 weeks prior to sample collection.

This stratification enabled comparative re-analysis of plasma fatty acid profiles across the three subgroups. No additional interventions or sample collections were performed. No other lipid-lowering drugs were administered, such as ezetimibe, fibrates, or PCSK9 inhibitors. A medical history of statin therapy lasting at least 8 weeks was documented prior to the blood plasma sampling.

### 2.3. Data Processing and Statistics

Model development, validation, and reporting adhered to the TRIPOD statement and included penalized variable selection (LASSO), stratified k-fold cross-validation, and bootstrap internal validation [43].

Statistical analysis was performed using R statistical software, version 4.4.2 and GraphPad Prism 8.0.1 (GraphPad Software, Boston, MA, USA), following the same methodology as in our previous study [40]. Categorical variables were represented as frequencies (%), whereas continuous variables were represented in mean (standard deviation)/median. Data distribution was assessed using the Shapiro–Wilk test. Two comparison frameworks were used: (i) Control (*n* = 50) vs. pooled Atherosclerosis (*n* = 52); and (ii) four-group comparisons (Control, *n* = 50; Atherosclerosis with atorvastatin, n = 19; Atherosclerosis with rosuvastatin, *n* = 21; and Atherosclerosis without statin therapy, *n* = 12). For four-group comparisons, one-way ANOVA was used for normally distributed outcomes with homogeneous variances; Welch’s ANOVA was applied when variances were unequal. For non-normally distributed outcomes, the Kruskal–Wallis test was used. When an omnibus test was significant, pairwise post hoc comparisons were conducted using Tukey’s HSD (after ANOVA) or Dunn’s test (after Kruskal–Wallis), with Holm-adjusted *p*-values to control for multiple testing within each family of pairwise comparisons. For two-group comparisons, Welch’s *t*-test was used for approximately normal outcomes and the Mann–Whitney U test otherwise. Effect sizes for between-group comparisons were quantified using Cliff’s delta (Δ) with 95% confidence intervals. Cliff’s delta was selected because it is a distribution-free, rank-based effect size that remains valid under non-normality and unequal group sizes, which were present in our cohort.

Sample size considerations were evaluated using Cohen’s f. An a priori power analysis for the primary two-group comparison (Control vs. pooled Atherosclerosis) assumed f = 0.40, α = 0.05, and power = 0.90 (Table S3). This corresponds to a standardized mean difference of approximately d ≈ 0.8 and was chosen as a conservative assumption relative to the observed variability in our key indices. For transparency, an achieved (post hoc) power calculation for the four-group setting was also performed using the same f = 0.40 and is reported in Table S3.

Correlation analyses were performed to assess relationships between fatty acid levels and lipid biomarkers within each statin subgroup. A two-sided *p*-value < 0.05 was considered statistically significant. Given that multiple biomarkers/ratios were evaluated in parallel (including multiple receiver operating characteristic (ROC) analyses), we additionally controlled for multiplicity within each set of related hypotheses using the Benjamini–Hochberg false discovery rate (FDR, q = 0.05). A two-sided *p*-value < 0.05 was considered statistically significant. 

ROC analyses and cut-off selection were performed using the cutpointr R package. The optimal decision threshold was chosen by maximizing Youden’s J; when multiple thresholds achieved the same maximum, the mid-point of the plateau was used. AUCs are reported with standard errors and 95% confidence intervals estimated using the DeLong method. Confidence intervals for sensitivity and specificity at the selected cut-off were calculated using the Wilson (Brown) method. The ROC-analysis for parameters and cut-off values for each associated with the best sensitivity and specificity were provided via the cutpointr R package. The validation of diagnostic power of chosen markers and indices was carried out by Stratified k-fold 10-fold Cross-Validation combined with 1000 bootstrap iterations due to low number of samples to form statistically precise validation set (70/30 scheme) for reliable assessment of discrimination or calibration.

Internal validation of diagnostic performance (both indices and the multivariable logit model) used stratified 10-fold cross-validation combined with 1000 bootstrap resamples to obtain optimism-corrected estimates of discrimination (AUC) and calibration (Brier score and calibration slope).

## 3. Results

### 3.1. FA Profile of Blood Plasma Stratified by Statin Therapy

According to the previous study [40], specific saturated and monounsaturated omega-9 and omega-3, and omega-6 polyunsaturated fatty acids (PUFAs) and their ratios (Table 1) were selected to evaluate changes in the activity of desaturase and elongase enzymes under atherosclerotic conditions. The table also presents the characteristics of the new metabolic Omega-6/3 Balance Index, detailed information about which will be provided in Section 3.3.

Out of the saturated and monounsaturated fatty acids, the current study focused on stearic acid (C18:0) and oleic acid (C18:1n-9) which had intergroup differences that were statistically significant. The concentrations of stearic acids were significantly lower in the atherosclerosis (AS) group than in controls (*p* < 0.0001), while the concentrations of oleic acids were greater in the AS group (*p* < 0.0001); accordingly, the C18:0/C18:1n-9 ratio was reduced in AS (*p* < 0.0001).

This study also focused on arachidonic acid (C20:4n-6), a key omega-6 fatty acid [44]. Its levels were significantly lower in the atherosclerosis (AS) group compared to the control group (*p* = 0.0033). The study also examined its precursor, dihomo-γ-linolenic acid (C20:3n-6), which was also significantly lower in the merged AS group (*p* = 0.0303). The levels of adrenic acid (C22:4n-6), produced from arachidonic acid via the enzyme ELOVL2/5, were also evaluated. Although its levels did not differ significantly between groups (*p* = 0.2977), the ratio of C20:4n-6 to C22:4n-6 was significantly lower in the AS group (*p* = 0.0006). In contrast, the ratio of C20:3n-6 to C20:4n-6 showed no significant difference between the groups (*p* = 0.8769).

All three omega-3 fatty acids (EPA, DPA, and DHA) were of interest in this study. EPA levels were significantly lower in the atherosclerosis (AS) group compared to the control group (*p* = 0.0159). In contrast, the levels of DPA (*p* = 0.1115) and DHA (*p* = 0.0692) did not differ significantly between groups. However, both the EPA/DPA ratio (*p* = 0.0371) and the EPA/DHA ratio (*p* = 0.0012) were significantly lower in the AS group. The DPA/DHA ratio showed no significant difference. 

Then, the plasma concentrations of C18, C18:1n-9 MUFA, omega-6 (C20:3n-6, C20:4n-6, and C22:4n-6), and omega-3 (EPA, DPA, and DHA) key PUFAs and their ratios were evaluated across four groups: healthy controls, and patients with carotid atherosclerosis treated with atorvastatin or rosuvastatin, or receiving no statin therapy.

In the subgroup analyses across four cohorts, stearic acid (C18:0; SFA) was lower in each patient subgroup than in controls (Control vs. atorvastatin *p* = 0.0024; Control vs. rosuvastatin *p* = 0.0006; and Control vs. no statin *p* = 0.0010), with no differences among the three patient subgroups (all *p* > 0.99). By contrast, oleic acid (C18:1n-9; MUFA) was higher in each patient subgroup than in controls (*p* < 0.0001 for atorvastatin and rosuvastatin; *p* = 0.0047 for no statin), again with no differences among the patient subgroups (*p* ≥ 0.58). Consistently, the C18:0/C18:1n-9 ratio was higher in controls than in all patient subgroups (all *p* < 0.0001) and did not differ among the patient subgroups (*p* ≥ 0.68).

For omega-6 PUFAs, the level of C20:3n-6 showed a non-significant downward trend in all atherosclerosis subgroups compared to controls. No group differences reached statistical significance (*p* > 0.05), though the mean % value in the rosuvastatin group was the lowest among all groups. In contrast, the levels of C20:4n-6 (arachidonic acid) were significantly lower in the atorvastatin (*p* = 0.0342) and rosuvastatin (*p* = 0.0080) groups compared to controls, whereas patients without statins did not differ significantly. For C22:4n-6 (adrenic acid), no significant intergroup differences were detected.

Among the omega-3 PUFAs, the EPA levels were significantly reduced in both the rosuvastatin (*p* = 0.0488) and no statin (*p* = 0.0224) groups relative to controls, but not in the atorvastatin group. DPA levels were significantly lower only in the no-statin group (*p* = 0.0302). DHA was significantly lower in the no-statin group compared to controls (*p* = 0.0334), but not in those on statins.

Regarding the pathway activity ratios, the C20:4n-6/C22:4n-6 ratio was significantly lower in all three atherosclerosis subgroups compared to controls (*p* < 0.05 for all), suggesting the reduced elongation efficiency of arachidonic acid. The C20:3n-6/C20:4n-6 ratio did not differ significantly between groups. In omega-3-related indices, the EPA/DHA ratio was significantly lower in all atherosclerosis subgroups (*p* < 0.05 for all comparisons), and the EPA/DPA ratio was significantly lower only in the atorvastatin group (*p* = 0.0290). The DPA/DHA ratio did not differ significantly in most comparisons, except for a modest but significant increase in the atorvastatin group versus rosuvastatin (*p* = 0.0206).

### 3.2. Evaluation of Potential Diagnostic Parameters

Despite the statistically significant differences observed between the control and AS groups in the individual markers (Table 1), these compounds cannot be used as a single diagnostic parameter, as their levels in blood plasma can vary widely in population. In order to compensate for these discrepancies, it is common practice to apply diagnostic indices that combine multiple individual metrics associated with one or more metabolic pathways (Table 1) [45]. Therefore, the diagnostic performance of the fatty-acid-based metrics differing across treatment groups were tested in a receiver operating characteristic analysis (ROC-analysis) (Table 2). For comparison, we took widely used parameters like the Omega-3 Status (sum of EPA and DHA to total fatty acids, %), AA/EPA, and omega-6/3 ratios (total omega-6/total omega-3 fatty acids) [9], and the most statistically significant metabolic indices from our study—the C18:0/C18:1n-9 and C20:4-n6/C22:4n-6 ratios and the new Omega-6/3 Balance Index, described in detail in Section 3.3. Below, area under the ROC curve (AUC) values are interpreted according to conventional cut-points (poor discrimination < 0.60; acceptable 0.60–0.69; and good ≥ 0.70).

The C18:0/C18:1n-9 ratio was found to have a stable and confident discriminatory ability in all pairwise tests. Compared to atorvastatin, the AUC was 0.835 (SE = 0.048; 95% CI = 0.740–0.929; *p* < 0.0001) versus a comparison with rosuvastatin AUC of 0.858 (SE = 0.046; 95% CI = 0.768–0.949; *p* < 0.0001), which has the most significant separation power. The control and the no-statin group also performed well with an AUC = 0.831 (SE = 0.065; 95% CI = 0.704–0.958; *p* = 0.0006). Together, these results demonstrate that the data show relatively narrow confidence intervals, which contribute to the strength and prospective clinical applicability of the C18:0/C18:1n-9 ratio as a discriminative biomarker.

Among PUFA-related indices, only the arachidonic acid to adrenic acid ratio (C20:4 n-6/C22:4 n-6) demonstrated good discrimination between the pair of Atorvastatin-treated vs. Control groups (AUC = 0.741 ± 0.061; 95% CI 0.622–0.861; *p* = 0.002), indicating a 74% probability that this index correctly ranks a case above a non-case. The Omega-3 Status, AA/EPA, and omega-6/omega-3 ratios yielded poor-to-acceptable AUCs (0.548–0.602) with non-significant *p*-values, suggesting limited utility in this subgroup. 

In Rosuvastatin patients compared to the Control group, the C20:4n-6/C22:4n-6 ratio again emerged as the strongest PUFA-based marker (AUC = 0.669 ± 0.069; 95% CI 0.532–0.805; *p* = 0.026), providing acceptable discrimination. Other indices remained in the poor-to-acceptable range (AUC 0.595–0.638) and did not reach statistical significance (*p* > 0.05). In the absence of statin therapy, three metrics achieved good discrimination and statistical significance. The Omega-3 Status approached significance (AUC = 0.676; *p* = 0.069), suggesting a potential but weaker association.

Overall, among PUFA-related indices the C20:4n-6/C22:4n-6 ratio demonstrated the most consistent and robust diagnostic capacity across all treatment strata, with statistically significant AUCs in each group. Statin therapy appeared to attenuate the discriminatory power of several indices: while AA/EPA and omega-6/omega-3 were predictive in the non-statin cohort, their performance diminished under atorvastatin and rosuvastatin. These findings suggest that statin exposure modulates the lipidomic profiles relevant to the outcome studied, and that C20:4n-6/C22:4n-6 may serve as a statin-resistant biomarker, but most of AUCs underscore the need to develop more sensitive, statin-resistant lipid indices to achieve reliable diagnostic discrimination, especially in statin-treated groups.

The diagnostic power of ratios, standard indices, and logit function for the group comparison were evaluated by Cliff’s Δ estimation (Table 3) [46]. The results of Cliff’s Δ for the comparison between the united Atherosclerosis group and Control could be found in Table S4.

The most significant positive effect of Cliff’s Δ was consistently observed for C18:0/C18:1n-9 when comparing the control group with the patient groups. The highest values were observed for the control group when compared with atorvastatin (0.71 (0.53–0.87)) in C18:0/C18:1n-9. The Omega-3 Status and C20:4-n6/C22:4n-6 values demonstrated a modest yet consistent positive association between the control group and the patient groups. It is notable that the value of Cliff’s Δ between patients and the control group for the Omega-3 Status exhibited an inverse pattern, with the most significant difference observed between the control group and the group of patients not treated with statins. The same pattern could be noted for omega-6/3. The biggest but negative differences between patient groups were found between the pairs of the No-statin group vs. Atorvastatin group and the No-statin group vs. Rosuvastatin group in the omega-6/3 ratio and AA/EPA. In contrast, other ratios in AA/EPA and omega-6/3 demonstrated only minor to moderate effects, with Cliff’s Δ values ranging from −0.09 to −0.26, and confidence intervals frequently including zero.

The result of the analysis could be summarized as follows: substantial Cliff’s Δ are predominantly attributable to contrasts between healthy controls and patients, with a minimal effect for AA/EPA and omega-6/3 and negligible disparities for other observed among treatment regimens, although the parameters for the smallest group comparisons for parameters C18:0, C18:1n-9, and C22:4n-6 (AdA) should be treated with caution (Table S3).

### 3.3. Novel Omega-6/3 Balance Index

A combinatorial assessment of C20:4n-6 (arachidonic acid, AA), C22:4n-6 (adrenic acid, AdA), EPA, DPA, and DHA was performed to capture the multi-step transformation within the omega-6 and omega-3 pathways and to take into account the high variability of EPA levels in the blood plasma [47]. The united formula with the inclusion of all three omega-3 fatty acids (EPA, DPA, and DHA) provided a balanced representation of the long-chain PUFA metabolism and reduced the influence of individual outliers. To further stabilize the variance, the square root of the multiplication of EPA and DPA concentrations was used. The final index integrated the ratios C20:4n-6/C22:4n-6, EPA/DHA, and DPA/DHA, which, together, reflect the activity of elongase (ELOVL2/5) and desaturase (FADS1/2) enzymes and demonstrated the best diagnostic performance.

All stages of model development and validation were conducted in accordance with the TRIPOD (Transparent Reporting of a multivariable prediction model for Individual Prognosis Or Diagnosis) statement to ensure methodological transparency and reproducibility.

The composite marker was designated the Omega-6/3 Balance Index (O6/3-BI) because it integrates several omega-3 and omega-6 intermediates that reflect desaturase- and elongase-dependent conversions rather than static fatty acid concentrations. This name emphasizes the biochemical specificity of the index while distinguishing it from simpler measures such as plasma EPA+DHA (Omega-3 Status) or the RBC Omega-3 Index.

The more effective index was from single FA percent concentrations (*Formula (1)*):

(1) $$Omega\text{-}6/3\;Balance\;Index\;=\;\frac{AA\times \sqrt{EPA\times DPA}}{AdA\;\times DHA}$$

The O6/3-BI was designed to reflect the most relevant long-chain PUFA conversion steps to inflammation resolution biology, with a focus on ≥C20 PUFAs that are SPM precursors [48]. Initially, we screened C20:3n-6, C20:4n-6, C22:4n-6, EPA, DPA, and DHA, and evaluated the pathway ratios that map to key enzymatic transformations. Only the components that remained statistically informative in our cohort were retained in the final index (C20:4n-6, C22:4n-6, EPA, DPA, and DHA). This resulted in an index structure combining the omega-6 elongation ratio C20:4n-6/C22:4n-6 with omega-3 progression towards DHA, thereby summarizing multi-step omega-6 and omega-3 pathway dynamics in a single measure.

For the omega-3 component, we calculated the geometric mean of EPA and DPA (√(EPA×DPA)) and divided it by DHA. This approach increases the robustness in plasma by reducing the sensitivity to short-term fluctuations in EPA levels [47], while retaining the pathway information by incorporating the EPA-to-DPA conversion into a single metabolic indicator related to DHA.

The calculation of the novel O6/3-BI values was conducted for all four groups of patients, and a comparison of the results with those of the control group was demonstrated (Table 1).

Healthy controls have the highest PUFA conversion “activity”, with an average index of 12.00 ± 4.94. All three atherosclerotic cohorts showed a statistically significant decrease in the levels of O6/3-BI. Compared with controls, the mean reductions were 30–34%, indicating the substantial impairment of long-chain PUFA inter-conversion in disease.

Cliff’s Δ confirmed large, positive shifts for both PUFAs, varying between the levels in Control vs. Atorvastatin, 0.44 (medium effect), and 0.56 (large effect) in Control vs. Rosuvastatin (Table 3). Whereas the treatment-to-treatment contrasts were small, the two groups Atorvastatin vs. Rosuvastatin have the borderline value of Δ between 0.14 which could be considered meaningful for future works related to a more in-depth assessment of the effect of statins on the blood fatty acid composition.

**Figure 1:**
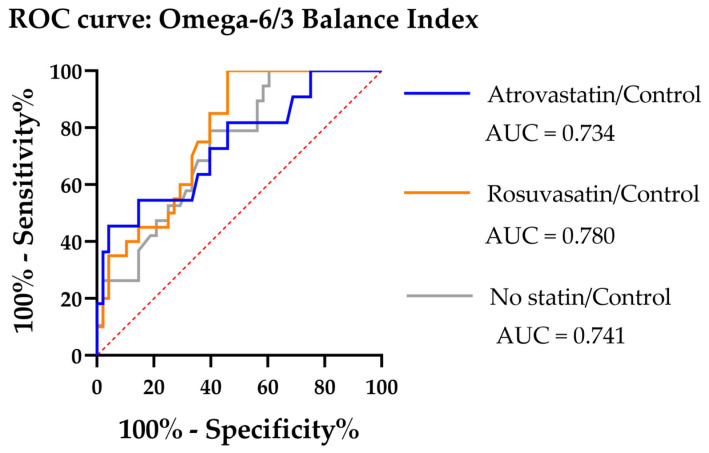
The receiver operating characteristic (ROC) curves for the O6/3-BI within every statin subgroup. Red dotted line is the reference line of no discrimination (random classifier; AUC = 0.5), where sensitivity equals 1−specificity.

Then, ROC curves for the novel O6/3-BI within every statin subgroup to see how well individual values separate atherosclerosis from the control were built (Table 2 and Figure 1).

The O6/3-BI demonstrates robust, statin-resistant diagnostic activity, maintaining good individual-level discrimination irrespective of atorvastatin, rosuvastatin, or no statin exposure. This consistency positions it as a promising biomarker for cardiovascular risk stratification and supports its further validation in larger, prospective cohorts. Therefore, the optimal cut-off was found by Youden’s J method (Table 4) [49,50,51]. The raw group differences showed practical diagnostic power and reveal that atorvastatin and rosuvastatin weakened accuracy. The tie-break rules were chosen as the mid-point (mean/median) of the plateau, neither FP nor FN clearly dominates in impact, and a reproducible rule that is robust to small numeric noise was formed and maximized specificity and sensitivity (Table 4). The ROC parameters for other FA and standard indices are placed in Table S5. Additional information about O6/3-BI’s cut-offs performances is also provided in Table S7.

The current cut-offs demonstrated that the no-statin group, due to the low number of participants, has a lower sensitivity, but also a lower value of the balanced cut-off. The united atherosclerosis group has similar values of sensitivity and accuracy to the group which undergoes therapy with rosuvastatin. Thus, in both statin-treated subgroups (atorvastatin and rosuvastatin), the index operated mostly as a highly sensitive but not very specific marker. Overall, the results showed that the cut-offs within the statin groups were higher and closer to the median value of the healthy group, and were therefore less accurate. These tendencies demonstrate that the O6/3-BI is suitable as a supporting biomarker but not as a stand-alone diagnostic tool.

The cut-off levels for the biggest pair of groups, control–atherosclerosis groups, also underwent the nested cross-validation (CV) procedures with the 1000 bootstrap approach to evaluate the stability of results (Table S8). The CV performance was not optimal; therefore, the logit function was formed.

### 3.4. Logit Diagnostic Function Based on SFA/MUFA Ratio and Omega-6/3 Balance Index

To elicit better CV performance while preserving the ability to distinguish atherosclerosis in the presence of statin therapy, we developed a parsimonious multivariable logistic (logit) diagnostic function. The Omega-6/3 Balance Index (O6/3-BI) was used as a candidate predictor along with five other diagnostic parameters (the Omega-3 Status, omega-6/3, AA/EPA, C20:4n-6/C22:4n-6, and C18:0/C18:1n-9). To have the right level of model complexity, we evaluated the feasibility with the Riley criteria and penalized selection (LASSO) (Table S6). We used the Riley criteria to make sure that the sample size and event rate of the proposed multivariate logistic model were sufficient and limited the complexity of the model and chances of overfitting, prior to the execution of the LASSO-based selection. Since the atherosclerosis subgroups of statins (atorvastatin, rosuvastatin, and no statin) were too small to allow us to model them in a stable subgroup-specific multivariate, we used the pooled atherosclerosis cohort for a pilot model development. Preserving the article’s focus on statin therapy to develop a function based on markers allowed us to separate all statin-treated groups from controls (Table 1, Table 2, Table 3 and Table 4) and could be viewed as a promising function for larger future studies of fatty acids’ profiles under statin treatment. 

Thus, the pooled dataset was used to develop the pilot model only for the biggest groups: the group of all atherosclerotic patients (*n* = 52) (statin-treated and non-treated) were merged into one outcome group and compared to the controls (*n* = 50) (Formula (2) and Table 5).

(2) $$logit\;\left(p\right)=5.254-0.145\times O6/3\text{-}BI-11.544\times (C18:0/C18:1n\text{-}9),$$

where p= 1(1+e−logitp)

In the final multivariable logistic regression model, only a limited subset of predictors retained independent associations with the outcome. The C20:4 n-6/C22:4 n-6 ratio did not contribute meaningfully to the model (β = 0.000), indicating the absence of independent predictive value after adjusting for other covariates. In contrast, the Omega-6/3 Balance Index showed a negative standardized β coefficient (β = −0.622; original scale β = −0.145), suggesting that higher values of this index were associated with a reduced probability of the outcome. The most prominent predictor was the C18:0/C18:1n-9 ratio, which demonstrated the strongest negative association (standardized β = −1.416; original scale β = −11.544), pointing to a substantial inverse relationship with the dependent variable. Overall, these findings highlight that the diagnostic performance of the model is driven primarily by the Omega-6/3 Balance Index and, more strongly, by the C18:0/C18:1n-9 ratio, while the other examined parameters did not contribute significantly.

The overall performance of the logistic regression model is summarized in the following Table 6, presenting the measures of discrimination, accuracy, explained variance, and calibration.

The model performance metrics indicated good overall discrimination and calibration. The AUC was 0.880, reflecting the excellent discrimination between outcome groups. The Brier score of 0.137 suggests the acceptable overall accuracy of the predicted probabilities. The good model fit was further supported by pseudo-R^2^ values (Cox–Snell R^2^ = 0.423, Nagelkerke R^2^ = 0.564). The calibration assessment showed minimal miscalibration, with a slope close to one (1.122), indicating that the predicted risks were neither systematically over- nor under-estimated across the range of probabilities.

**Figure 2:**
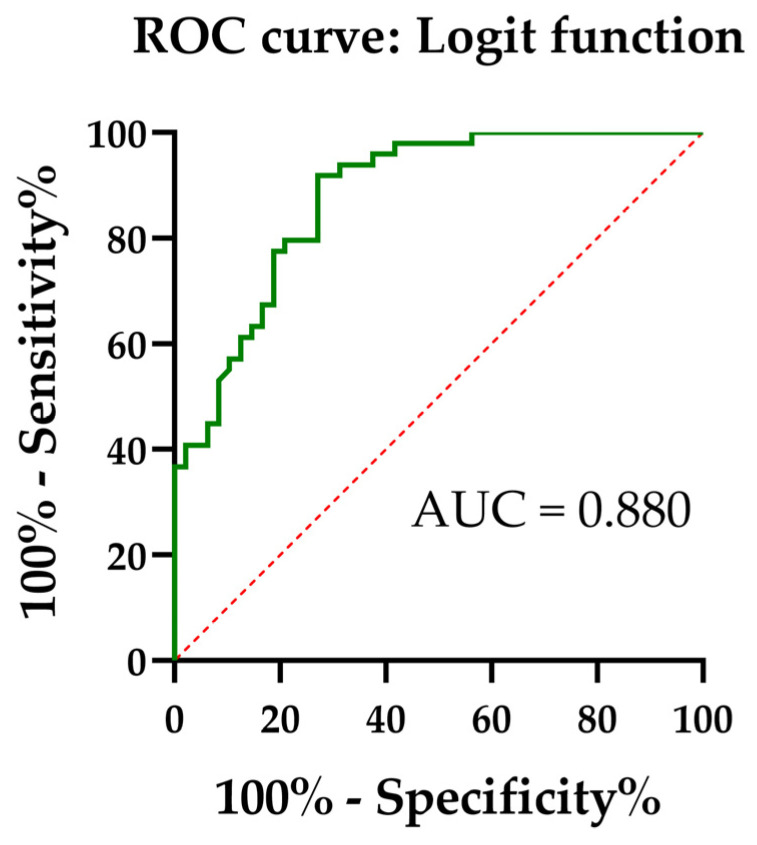
ROC curves for logit function for two groups—Control and Atherosclerosis (all atherosclerosis patients pooled, including atorvastatin, rosuvastatin, and no-statin subgroups). Red dotted line is the reference line of no discrimination (random classifier; AUC = 0.5), where sensitivity equals 1-specificity.

The ROC curve, Cliff’s Δ result, and cut-off of the logit function demonstrated a high discriminative ability (Figure 2, Table 7), clearly above the reference line of no discrimination and better than previous biomarkers. This indicates that the model provides exceeding accuracy in distinguishing between the study groups. The final logistic diagnostic model demonstrated excellent and reproducible discrimination under repeated stratified cross-validation with bootstrap correction (Table S8).

The model achieved an AUC of 0.880 (95% CI: 0.811–0.941) and an overall accuracy of 78.9%, at the optimal Youden cut-off of 0.02 on the logit scale (corresponding to a predicted probability of *p* = 0.505). The Matthews correlation coefficient (MCC = 0.62) indicated a moderate-to-good agreement between the predicted and observed classifications, consistent with the earlier Cohen’s κ value of 0.57 (Tables S7 and S8). The confusion matrix (TN = 35, FP = 13, FN = 8, TP = 42) confirmed a balanced distribution of misclassifications, with no systematic bias (McNemar *p* = 3.83 × 10^−1^). The model’s accuracy remained significantly higher than the no-information rate (*p* < 0.0001), confirming the non-random predictive performance (Table S8).

Overall, the solo O6/3-BI showed significant differences between the patient groups, although the diagnostic performance considering the CV was not optimal. However, the logit function demonstrated a higher stability and sensitivity when additional C18:0/C18:1n-9 data was included.

## 4. Discussion

Our study highlights the potential value of the SFA/MUFA-based ratio in discriminating between atherosclerotic and healthy individuals. More importantly, this parameter may help identify those with persistent metabolic alterations that remain despite statin therapy. Among all tested indices, the C18:0/C18:1n-9 ratio showed the strongest individual diagnostic performance, with excellent discrimination across all comparisons (AUC 0.831–0.858, *p* < 0.001). This ratio reflects the SCD1 activity, which plays a key role in fatty acids’ profile alteration, hepatic lipogenesis, and metabolic homeostasis. Despite the absence of significant variation in relation to statin subtypes, the consistently lower values observed across all patient groups indicate that C18:0 desaturation may be a pivotal contributor to atherosclerosis. These findings are consistent with prior studies, which have connected a high SCD1 activity with lipogenesis and insulin resistance in metabolic syndrome, both of which are tightly linked to atherosclerosis development [27,29].

In summary, the consistently lower C18:0/C18:1n-9 ratio found in all atherosclerosis groups compared to healthy controls supports using this parameter as a potential metabolic indicator in future studies evaluating the broader effects of different lipid-lowering therapies.

Our study results show that atherosclerosis, particularly in patients receiving statin therapy, is associated with noticeable changes in the plasma polyunsaturated fatty acid (PUFA) composition. Mainly, the changes involve fatty acids that are products of the desaturation and elongation pathways. While the levels of dihomo-γ-linolenic acid (C20:3n-6) did not differ significantly between groups, the consistent downward trend across all atherosclerosis subgroups suggests a reduction in early omega-6 metabolic activity. This trend is consistent with previous studies indicating that C20:3n-6, a precursor of series-1 prostaglandins (notably PGE1) with anti-inflammatory properties, is susceptible to oxidative stress and inflammation, both of which can disrupt Δ6-desaturase activity [52].

A significant reduction in arachidonic acid (AA, C20:4n-6) levels was found in statin-treated patients. Arachidonic acid is an essential component of cardiovascular biology and a pro-inflammatory mediator [44,53]. These data are consistent with the findings of another study which demonstrated a decrease in arachidonic acid levels in rats treated with statins [54]. This suggests that decreased AA levels in this context may reflect impaired synthesis from linoleic acid rather than increased utilization for the production of specialized mediators during inflammation. This reinforces the hypothesis that statins, especially rosuvastatin in our cohort, can disrupt the activity of elongase (ELOVL5) and desaturase (FADS1) enzymes involved in the synthesis of arachidonic acid precursors [55]. Nevertheless, it should be noted that the published data on arachidonic acid levels are inconsistent. Lipidomic profiling in large human cohorts has shown that statins tend to reduce the absolute concentration of polyunsaturated fatty acids (PUFAs), particularly omega-6 species, while the percentage composition of individual fatty acids within the total fatty acid pool often remains relatively stable [56]. Several studies have reported elevated AA concentrations in patients with atherosclerosis, indicating that AA dynamics may vary depending on disease stage, sample type, or methodological differences [41].

Notably, the levels of adrenic acid (C22:4n-6), which is formed by the elongation of arachidonic acid, did not show significant changes. Despite this, the ratio of C20:4n-6 to C22:4n-6 was significantly reduced across all patient groups. This can indicate a decrease in elongation activity, which cannot be observed when studying the absolute levels only. Consequently, the C20:4n-6/C22:4n-6 ratio may be a more sensitive measure of the ELOVL2 and ELOVL5 activity than the levels of adrenic acid.

Among the omega-3 fatty acids, eicosapentaenoic acid (EPA) experienced the most significant reduction, particularly in patients taking rosuvastatin and those not taking statins. EPA is a critical precursor for the biosynthesis of E-series resolvins, a family of lipid mediators that promote the resolution of inflammation. Reduced EPA levels may therefore impair the body’s ability to resolve vascular inflammation effectively [57,58].

Previous studies have indicated that persistent vascular inflammation can last in statin-treated patients with low omega-3 levels [59,60].The clinical trials indicate that adding high-purity EPA (icosapent ethyl) to statin treatment reduces cardiovascular events. However, the impact on inflammatory biomarker levels and other omega-3 formulations is not as consistent [61] The combination of the findings suggests that omega-3 supplementation should be considered as an addition to statin therapy to help minimize residual vascular inflammation, particularly for individuals with low omega-3 levels [54,62].

It is noteworthy that, in our study, rosuvastatin was linked with a more pronounced decrease in EPA levels in comparison to atorvastatin. In the published literature, several statins, including atorvastatin and rosuvastatin, have been linked to reductions in circulating EPA and DHA in some cohorts. However, the results vary by population and sample matrix. There is limited head-to-head evidence that rosuvastatin decreases EPA more than atorvastatin, so this comparison should be interpreted with caution [63]. Rosuvastatin is usually more effective than atorvastatin in lowering LDL-C and has a powerful hepatic effect, which may play a role in the changes in fatty acid metabolism [64].

The significantly lower EPA/DHA ratio in all atherosclerosis groups suggests an alteration in the normal relationship between omega-3 precursors and their longer-chain product [65]. This ratio is often used as indicators of omega-3 fatty acid conversion efficiency and reflect the activity of desaturation and elongation pathways [66]. The consistent reduction in these ratios suggests that either EPA synthesis is reduced or EPA is being preferentially metabolized, potentially to produce anti-inflammatory mediators. More generally, lower EPA could plausibly limit the capacity to generate pro-resolving signals in the vascular wall. This mechanistic interconnection is well-supported, but the clinical implications are conditional on diet, matrix, and genetics [67].

There were no significant differences in the DPA-to-DHA ratio in most of the comparisons except that in the atorvastatin group compared to the rosuvastatin group. The increase in the DPA-to-DHA ratio was modest but statistically significant (*p* = 0.0206). Because docosapentaenoic acid (DPA) is a transitional factor in the omega-3 metabolic pathway between eicosapentaenoic acid (EPA) and docosahexaenoic acid (DHA), an elevated DPA/DHA ratio may indicate a slower conversion of DPA to DHA [68]. In our sample, the difference between the groups of patients treated with atorvastatin and rosuvastatin indicates that the two drugs might have slightly different effects on the enzymes in this pathway. The observed increase in the DPA/DHA ratio in the atorvastatin group could thus indicate a weaker effect of atorvastatin on this enzyme system that than of rosuvastatin. Nevertheless, the recent experimental data demonstrate that statins may regulate and, in some cases, induce FADS and ELOVL expression instead of suppressing them, as a matter of fact [22,41], so these interpretations should be considered exploratory.

Collectively, our results support the hypothesis that statins have therapeutic effects by not only reducing low-density lipoprotein cholesterol (LDL-C), but also affecting the metabolism of polyunsaturated fatty acids (PUFAs) [63]. We observed ongoing disturbances in PUFA conversion among patients with atherosclerosis, including those not undergoing therapy and those taking statins. However, the statin-treated group exhibited additional statistically significant metabolic shifts beyond those observed in the untreated group. These imbalances involve disrupted fatty acid ratios and a reduced conversion efficiency in both the omega-3 and omega-6 pathways.

To have a more accurate evaluation of these metabolic changes, this work presents the Omega-6/3 Balance Index. In contrast to static measurements of individual fatty acid levels, this index reflects the functional activity of key metabolic enzymes and the flow of substrates through the desaturation and elongation pathways. It could provide a more sensitive and dynamic indication of lipid metabolism in atherosclerotic patients.

We compared conventional indices and ratios, including the Omega-3 Status (EPA + DHA), omega-6/omega-3 ratio, and AA/EPA ratio, as well as the ratios of the pathways evaluated in this work [9]. Conventional indices in blood plasma demonstrated a poor to acceptable diagnostic performance, with AUCs generally within the 0.55–0.64 range and being insignificant *p*-values in the atorvastatin and rosuvastatin subgroups. Thus, the discriminatory ability of these parameters is reduced with statin therapy.

In comparison, the ratio of arachidonic acid to adrenic acid (C20:4n-6/C22:4n-6) was found to give a consistent and statistically significant discrimination across all patient groups with AUC values of 0.669 in rosuvastatin patients with *p* = 0.026 to 0.741 in atorvastatin patients with *p* = 0.002. This indicates that the elongation process that is mainly regulated by ELOVL2 and ELOVL5 is a better indicator of PUFA metabolism compared to absolute fatty acid concentrations.

The poor diagnostic capabilities of conventional PUFA-related indices in plasma can be caused by several factors related to statin treatment and the nature of the plasma compartment. Statins can also regulate lipid metabolism, including desaturase activity and elongase, and global changes in these pathways can reduce the between-group differences in simple plasma ratios [41,69]. Plasma PUFA concentrations are also influenced by dietary intake, rapid turnover, and transport dynamics, making them less reflective of long-term metabolic changes or tissue-level enzyme activity [70]. As a result, ratios in plasma may appear “normal” even when the underlying synthesis or resolution pathways are disrupted. When combined with the poor diagnostic value of conventional fatty acid ratios in blood plasma, these results support the argument that more sensitive, statin-resistant lipidomic indices are necessary in order to measure the PUFA metabolism in cardiovascular disease populations accurately.

**Figure 3:**
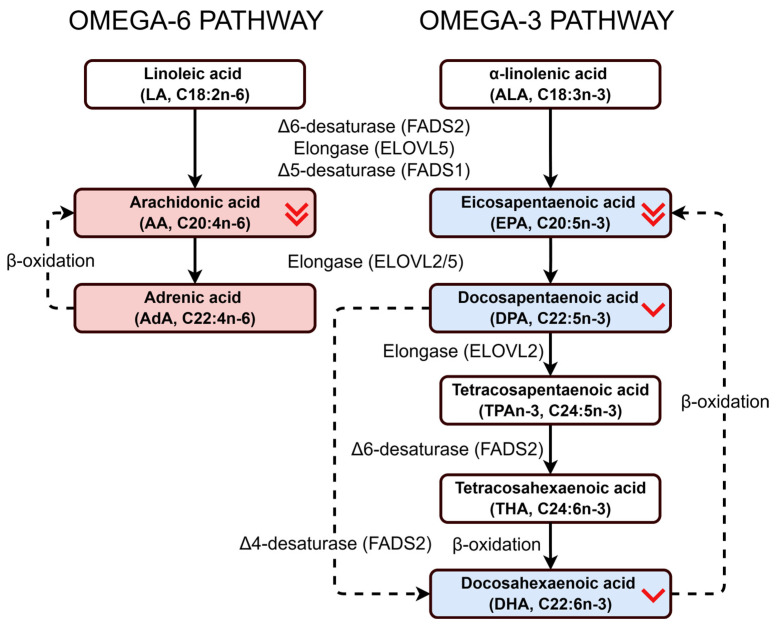
Schematic representation of the omega-6 (left) and omega-3 (right) long-chain polyunsaturated fatty acid (PUFA) pathways. Solid lines represent canonical Sprecher pathway reactions, including desaturation (Δ5-, Δ6-), elongation, and peroxisomal β-oxidation steps forming arachidonic acid (AA), adrenic acid (AdA), eicosapentaenoic acid (EPA), docosapentaenoic acid (DPA), and docosahexaenoic acid (DHA). Dotted lines indicate the Δ4-desaturase route, which is currently under investigation as a potential alternative pathway for direct DPA→DHA conversion, as well as retroconversion steps (AdA→AA and DHA→EPA) through peroxisomal β-oxidation. Intermediate products between the initial omega-6 (linoleic acid) and omega-3 (α-linolenic acid) precursors and the key long-chain metabolites (AA and EPA) are not shown for clarity, since only the compounds relevant to this study are depicted. Omega-6 fatty acids included in the Omega-6/3 Balance Index (AA and AdA) are shown in red, and omega-3 fatty acids (EPA, DPA, and DHA) in blue. A double red indicates a statistically significant decrease in selected fatty acid levels in two AS subgroups compared to the control group, while a single arrow indicates a decrease in one AS subgroup. However, there is a downward trend in all the fatty acids indicated by arrows (see Table 1). These interconnected reactions represent the biochemical basis for the Omega-6/3 Balance Index (O6/3-BI), which integrates AA, AdA, EPA, DPA, and DHA, to reflect multi-step enzymatic conversion of long-chain fatty acids [11,36,71,72].

Therefore, we further conducted a combinatorial analysis of the indicators C20:4n-6 (AA), C22:4n-6 (adrenic acid, AdA), EPA, DPA, and DHA, selecting the best combination in terms of diagnostic parameters. As a result, the formula included the metabolic ratios C20:4n-6/C22:4n-6, EPA/DHA, and DPA/DHA, reflecting changes in the activity of the ELOVL2/5 and FADS1/2 enzymes (Figure 3).

The selected fatty acids are key products of fatty acid metabolism and act as precursors to a variety of physiologically active lipid mediators. These include prostaglandins, thromboxanes, and leukotrienes derived from AA; resolvins of the E-series derived from EPA; resolvins of the D-series, protectins and maresins derived from DHA; and lipoxins derived from AA and EPA [57,73,74,75]. Furthermore, AA is a substrate of endocannabinoids, such as anandamide (AEA) and 2-arachidonoylglycerol (2-AG). EPA and DHA undergo parallel reactions to produce ethanolamide analogs, including eicosapentaenoyl ethanolamide (EPEA) and docosahexaenol ethanolamide (DHEA), that have anti-inflammatory and neuroprotective effects [76,77]. These mediators have a regulatory effect on inflammation, vascular tone, immune activity, and neural signaling. Finally, C22:4n-6 (adrenic acid, AdA) is the downstream elongation product of AA and may be used as a marker of disruptions in the metabolism of arachidonic acid [78]. Adrenic acid is produced from arachidonic acid through elongation and can also be retroconverted back to AA by peroxisomal β-oxidation [36]. This reversible step helps maintain a stable pool of arachidonic acid for the synthesis of signaling molecules such as prostaglandins and leukotrienes. Thus, AdA acts as a metabolic reservoir that supports continuous AA availability, and the C20:4n-6/C22:4n-6 ratio reflects the balance between elongation and retroconversion in the omega-6 pathway.

The combination of parameters with the highest discriminatory power between the studied groups and the established clinical relevance resulted in the development of the Omega-6/3 Balance Index. The term ‘Omega-6/3 Balance Index’ (O6/3-BI) emphasizes the composite nature of the newly proposed index, which combines the absolute levels of polyunsaturated omega-6 and omega-3 fatty acids with dynamic conversion through the desaturase and elongase pathways. The term ‘balance’ highlights the index’s integrative quality, combining multiple omega-3 and omega-6 components into a single score, and emphasizes the importance of maintaining an optimal balance between omega-3 and omega-6 PUFAs. The ‘Omega-6/3 Balance Index’ intuitively signals whether a patient’s PUFA profile leans towards pro-inflammatory omega-6 dominance or a healthier omega-3-rich state.

The Omega-6/3 Balance Index was empirically calculated to summarize the multi-step alteration of the omega-3 and omega-6 pathways. We included DPA alongside EPA and DHA because EPA, as an upstream precursor, demonstrates greater short-term plasma variability than DHA, particularly following ingestion and during the postprandial period [47]. Including an intermediate DPA in the index has been used to achieve two goals. First, it reduces the effect of outliers in the EPA dataset. Second, it retains pathway information by capturing the EPA → DPA → DHA segment rather than relying solely on EPA.

In order to limit the heteroscedasticity from highly skewed omega-3 distributions, a straightforward variance-stabilizing transform (the square root) of EPA × DPA was used, a standard approach for right-skewed biochemical values that improves robustness without distorting ranks [79,80]. The combination of partially correlated lipid markers into a single score is a concept that aligns with the best practices in biomarker research. In such studies, multi-marker models often exhibit enhanced discrimination compared to single indices [45,81].

Moreover, the proposed composite index was supported by the currently emerging evidence of the parallel biosynthesis of DHA. While the classical Sprecher pathway describes the elongation of EPA to DPA, further elongation to 24:5n-3, desaturation to 24:6n-3, and peroxisomal chain shortening to DHA, recent studies have highlighted an alternative route involving the direct Δ4-desaturation of DPA to DHA. Evidence from a number of vertebrate models has demonstrated that this pathway is more active than was previously assumed [71,82]. By including EPA, DPA, and DHA simultaneously, the Omega-6/3 Balance Index reflects the variability across both the “Sprecher pathway” and the Δ4-desaturase route, thereby reducing the noise associated with the lability of EPA in plasma and better reflecting the overall efficiency of the long-chain omega-3 metabolism [71,83].

To put it another way, the Omega-6/3 Balance Index is a comprehensive biochemical marker that reflects the combined activity of several enzymatic steps in fatty acid metabolism, including Δ4-, Δ5-, and Δ6-desaturases and elongases [84]. It is also linked to the major transcriptional regulators of lipid metabolism. For example, PPAR-α promotes long-chain fatty acid oxidation and can modulate PUFA profiles. At the same time, SREBP-1c regulates genes involved in lipid synthesis and affects the balance between long-chain saturated, monounsaturated, and polyunsaturated fatty acids [27,85]. The concept of integrating various pathways into a single metabolic signature, rather than using a single fatty acid index, has been mentioned in the literature [86,87,88,89]. However, until recently, no single formula has combined several PUFA ratios of pathways into a diagnostic index, highlighting the originality of the indices presented herein.

The Omega-6/3 Balance Index has reached its peak level in healthy participants, while being reduced by about 30% in participants with atherosclerosis, indicating a reduction in the metabolic flux rates of PUFA. The ROC has shown that this index has strong diagnostic power, with AUC measures ranging from approximately 0.73 to 0.78 across subgroups, and the differences are statistically significant according to the *p*-values. Notably, the index retained its effectiveness in patients subjected to statin treatment, but the traditional indices lost sensitivity. For instance, in patients not receiving statins, the Omega-3 Status, omega-6/3, or AA/EPA ratio was found to have acceptable predictive value. However, in statin-treated subgroups, their AUC values declined to approximately 0.55–0.64 and no longer reached statistical significance. This finding is consistent with prior reports, including Nozue et al.’s, which have shown that the progression of plaque in statin-treated patients depends on changes in the omega-3/omega-6 ratio. The only subjects that showed a regression of the atheroma were those who had obtained a better ratio, presumably due to dietary intake or supplementation [65]. Taken together, the findings of this study suggest that the Omega-6/3 Balance Index functions as a statin-resistant biomarker, integrating several enzymatic pathway activities and reflecting persistent metabolic disturbances that are less influenced by pharmacologic therapy.

To increase the discriminatory ability in addition to individual markers, we developed a parsimonious logistic regression (logit) model combining the SFA/MUFA ratio C18:0/C18:1n-9 and the Omega-6/3 Balance Index. The conceptual framework for developing such a combination diagnostic equation was based on Li et al. (2025), who demonstrated that adding several parameters to a single logit equation can significantly enhance the diagnostic accuracy [90]. The pooled dataset, the Controls and Atherosclerosis cases, without stratification by statin use, was used to construct the model because the sample sizes of the individual subgroups were too small to support a stable multivariable modeling and would increase the risk of over-fitting in accordance with TRIPOD and sample-size guidance [43,91]. A subgroup-specific multivariable model is an object of a future larger prospective cohort. Penalization (LASSO) was used to pre-screen predictor variables to minimize redundancy, and these were then placed into a multivariable logit. The last model included the C18:0/C18:1 ratio and Omega-6/3 Balance Index with negative coefficients, which means that a low value on either of the two variables must be linked to a higher likelihood of atherosclerosis (Table 5). This strategy aligns with the established practice whereby combining complementary biomarkers can yield higher AUCs than any single constituent [90,92].

Non-parametric methods were used to compare ROC curves, and decision thresholds were chosen by maximizing the Youden J statistics, and were presented with sensitivity, specificity, and 95% CIs. This approach is typical of converting continuous scores into practical decision rules [49,93].

The combined logit model, which was built on the C18:0/C18:1 ratio and the Omega-6/3 Balance Index, showed outstanding diagnostic results (AUC = 0.880, accuracy = 78.6%, sensitivity = 84.0%, and specificity = 72.9%). These values were obviously better than those found using the single indices: even the best single marker, C18:0/C18:1, had an AUC of 0.83–0.86, whereas the Omega-6/3 Balance Index alone had an AUC of 0.73–0.78.

Therefore, the combined equation performed better than each of the parameters individually, indicating the complementary biological data represented by the SCD1-mediated MUFA reorganization and the multi-step PUFA desaturation/elongation processes that are directly pertinent to atherosclerosis. External validation, decision-curve analysis, and pre-specified subgroup models per statin type will be possible in larger cohorts and are recommended by reporting guidelines [43,94].

### Limitations

This study has several limitations. The sample size was relatively small and conducted in one research center, which may limit generalizability. Both the Omega-6/3 Balance Index and the logit function were developed and internally validated within this dataset only. External validation in larger and independent cohorts is required to confirm their robustness. Plasma fatty acid measurements are influenced by recent diet, and the observational design may introduce confounding related to statin use and nutrition. Future studies should include dietary assessments, randomized treatment comparisons, and the prospective validation of these markers for cardiovascular risk prediction. Overall, the current work on the Omega-6/3 Balance Index and the logit function should be considered one example of the pioneering work in the assessment of atherosclerosis. However, future studies should validate these methods prospectively and evaluate their predictive value for cardiovascular outcomes.

## 5. Conclusions

The present study demonstrated that indices derived from PUFA and MUFA profiles are more effective diagnostic tools for atherosclerosis-related fatty acid disturbances than traditional indices and ratios. The lower Omega-6/3 Balance Index in atherosclerotic patients indicated significant alterations in the enzymatic activity of long-chain fatty acid metabolism. A decrease in the C18:0/C18:1n-9 ratio has been demonstrated to be a reliable indicator of a more general metabolic impairment. Notably, when combined in a logistic model, these two markers provided superior discrimination between atherosclerosis patients and healthy controls compared to any single index.

An important finding is that the Omega-6/3 Balance Index maintained its diagnostic value even in statin-treated patients, whereas conventional indices (e.g., the Omega-3 Status (EPA + DHA), AA/EPA, and omega-6/3) lost significance in this context. This statin-resistant behavior suggests that O6/3-BI, especially in combination with the C18:0/C18:1n-9 ratio, could serve as a reliable biomarker for monitoring atherosclerosis-associated metabolic alterations independent of LDL-cholesterol-lowering therapy. Our results highlight the potential of these lipidomic indices as next-generation cardiovascular risk markers that capture aspects of residual inflammatory risk.

Future research should validate these markers in larger cohorts and examine their longitudinal dynamics under different statin regimens and dietary backgrounds. In particular, it would be valuable to assess whether improving a patient’s omega-3 status (e.g., through dietary supplementation) favorably modifies these indices and translates into better clinical outcomes. Such studies will clarify whether the O6/3-BI and related indices not only predict risk but also guide personalized therapy. If confirmed, these fatty acid biomarkers could become valuable tools for risk stratification in preventive cardiology and for optimizing treatment in atherosclerotic patients receiving routine lipid-lowering treatment.

## Figures and Tables

**Table 1:** The mean molar percentage content of selected fatty acids and their ratios in the plasma of the four studied groups.

Parameters	Group, Number of Samples			
	Control, *n* = 50	Atherosclerosis, Atorvastatin, *n* = 19	Atherosclerosis, Rosuvastatin, *n* = 21	Atherosclerosis, No Statin, *n* = 12
**Selected FA: SFA, MUFA, omega-6, -3 pathways**				
C18:0	9.27 ± 1.83 ^**2,3,4**^	7.42 ± 0.93 ^**1**^	7.45 ± 0.96 ^**1**^	7.45 ± 1.01 ^**1**^
C18:1n-9	23.13 ± 3.38 ^**2,3,4**^	27.33 ± 2.75 ^**1**^	28.49 ± 3.49 ^**1**^	28.01 ± 3.66 ^**1**^
C20:3n-6	1.31 ± 0.31	1.18 ± 0.41	1.15 ± 0.29	1.20 ± 0.35
AA, C20:4n-6	7.53 ± 1.99 ^**2,3**^	6.56 ± 1.49 ^**1**^	6.19 ± 1.79 ^**1**^	6.67 ± 1.61
AdA, C22:4n-6	0.17 ± 0.06	0.19 ± 0.06	0.17 ± 0.04	0.19 ± 0.05
DPA, C22:5n-3	0.38 ± 0.10 ^**4**^	0.39 ± 0.12	0.34 ± 0.07	0.32 ± 0.06 ^**1**^
EPA, C20:5n-3	0.59 ± 0.32 ^**3,4**^	0.49 ± 0.26	0.45 ± 0.21 ^**1**^	0.41 ± 0.19 ^**1**^
DHA, C22:6n-3	1.97 ± 0.68 ^**4**^	1.84 ± 0.48	1.77 ± 0.39	1.66 ± 0.34 ^**1**^
**Indices and ratios**				
Omega-3 Status	2.70 ± 1.07 ^**3,4**^	2.33 ± 0.71	2.22 ± 0.50 ^**1**^	2.06 ± 0.50 ^**1**^
AA/EPA	13.80 ± 7.92	15.47 ± 8.01	16.14 ± 7.49	19.36 ± 7.99
Omega-6/3	11.63 ± 4.57 ^**4**^	13.23 ± 4.94	12.90 ± 2.86	14.05 ± 2.60 ^**1**^
C18:0/C18:1n-9	0.42 ± 0.13 ^**2,3,4**^	0.28 ± 0.05 ^**1**^	0.27 ± 0.06 ^**1**^	0.27 ± 0.07 ^**1**^
C20:4-n6/C22:4n-6	45.14 ± 12.84 ^**2,3,4**^	36.05 ± 5.46 ^**1**^	37.39 ± 8.64 ^**1**^	37.01 ± 9.46 ^**1**^
Omega-6/3 Balance Index	12.00 ± 4.94 ^**1**^	8.35 ± 2.56 ^**1**^	7.91 ± 2.05 ^**1**^	8.13 ± 3.32 ^**1**^

Note: Chosen parameters differ significantly (*p* < 0.05): **^1^**, **^2^**, **^3^**, **^4^** accordingly in comparison to data of control group, group with atherosclerosis taking atorvastatin, group with atherosclerosis taking rosuvastatin, and group with atherosclerosis undergoing treatment without statin. The values are given in Mean ± SD format.

**Table 2:** Area under the receiver operating characteristic curve (ROC curve) (AUC), 95% confidence intervals, and Wald test *p*-values for each lipid marker in the plasma of the three studied groups with atherosclerosis in comparison to control.

Group	Parameters	Omega-3 Status	AA/EPA	Omega-6/3	C20:4n-6/ C22:4n-6	C18:0/ C18:1n-9	Omega-6/3 Balance Index
Control–Atorvastatin	AUC	0.602	0.548	0.588	0.741	0.835	0.734
	Std. Error	0.069	0.077	0.073	0.061	0.048	0.063
	95% CI	0.467–0.738	0.396–0.698	0.445–0.731	0.622–0.861	0.740–0.929	0.611–0.857
	*p*-value	0.1926	0.5550	0.2620	0.0022	<0.0001	0.0030
Control–Rosuvastatin	AUC	0.638	0.595	0.626	0.669	0.858	0.780
	Std. Error	0.064	0.072	0.067	0.069	0.046	0.056
	95% CI	0.512–0.764	0.454–0.736	0.495–0.757	0.532–0.805	0.768–0.949	0.671–0.889
	*p*-value	0.0728	0.2206	0.1028	0.0262	<0.0001	0.0003
Control– No statin	AUC	0.676	0.701	0.711	0.698	0.831	0.741
	Std. Error	0.068	0.079	0.066	0.090	0.065	0.087
	95% CI	0.543–0.809	0.546–0.855	0.582–0.840	0.521–0.874	0.704–0.958	0.569–0.912
	*p*-value	0.0688	0.0391	0.0296	0.0419	0.0006	0.0134

**Table 3:** Cliff’s Δ (95% CI) for all pairwise group comparisons.

Indices and Ratios	Group	Control	Atorvastatin	Rosuvastatin	No Statin
Omega-3 Status	Control		0.21 (−0.07–0.48)	0.32 (0.06–0.56)	0.39 (0.12–0.64)
	Atorvastatin	0.21 (−0.07–0.48)		0.17 (−0.22–0.52)	0.24 (−0.19–0.68)
	Rosuvastatin	0.32 (0.06–0.56)	0.17 (−0.22–0.52)		0.20 (−0.24–0.65)
	No statin	0.39 (0.12–0.64)	0.24 (−0.19–0.68)	0.20 (−0.24–0.65)	
AA/EPA	Control		−0.09 (−0.40–0.20)	−0.19 (−0.48–0.10)	−0.40 (−0.69–0.09)
	Atorvastatin	−0.09 (−0.40–0.20)		−0.12 (−0.49–0.27)	−0.36 (−0.76–0.05)
	Rosuvastatin	−0.19 (−0.48–0.10)	−0.12 (−0.49–0.27)		−0.31 (−0.70–0.10)
	No statin	−0.40 (−0.69–−0.09)	−0.36 (−0.76–0.05)	−0.31 (−0.70–0.10)	
Omega-6/3	Control		−0.13 (−0.42–0.16)	−0.26 (−0.52–0.02)	−0.43 (−0.69–−0.17)
	Atorvastatin	−0.13 (−0.42–0.16)		−0.15 (−0.51–0.22)	−0.32 (−0.72–0.08)
	Rosuvastatin	−0.26 (−0.52–0.02)	−0.15 (−0.51–0.22)		−0.30 (−0.68–0.13)
	No statin	−0.43 (−0.69–−0.17)	−0.32 (−0.72–0.08)	−0.30 (−0.68–0.13)	
C20:4-n6/C22:4n-6	Control		0.46 (0.20–0.70)	0.37 (0.09–0.63)	0.38 (0.01–0.73)
	Atorvastatin	0.46 (0.20–0.70)		−0.04 (−0.40–0.34)	0.04 (−0.42–0.51)
	Rosuvastatin	0.37 (0.09–0.63)	−0.04 (−0.40–0.34)		0.01 (−0.43–0.47)
	No statin	0.38 (0.01–0.73)	0.04 (−0.42–0.51)	0.01 (−0.43–0.47)	
C18:0/C18:1n-9	Control		0.71 (0.53–0.87)	0.73 (0.54–0.89)	0.67 (0.40–0.90)
	Atorvastatin	0.71 (0.53–0.87)		0.13 (−0.23–0.50)	0.04 (−0.42–0.49)
	Rosuvastatin	0.73 (0.54–0.89)	0.13 (−0.23–0.50)		−0.03 (−0.46–0.40)
	No statin	0.67 (0.40–0.90)	0.04 (−0.42–0.49)	−0.03 (−0.46–0.40)	
Omega-6/3 Balance Index	Control		0.44 (0.17–0.66)	0.56 (0.29–0.75)	0.48 (0.09–0.76)
	Atorvastatin	0.44 (0.17–0.66)		0.14 (−0.24–0.49)	0.10 (−0.40–0.54)
	Rosuvastatin	0.56 (0.29–0.75)	0.14 (−0.24–0.49)		0.01 (−0.46–0.49)
	No statin	0.48 (0.09–0.76)	0.10 (−0.40–0.54)	0.01 (−0.46–0.49)	

Note: The green color saturation demonstrates the magnitude of Cliff’s Δ. The saturation of green color shows the positive values, the yellow color shows the negative values of delta, and the grey color shows the empty cells.

**Table 4:** Diagnostic performance and three cut-off-Youden (mid-plateau rule), and maximum sensitivity and specificity for O6/3-BI between four groups (pairs Control–Atorvastatin; Control–Rosuvastatin; Control–No statin) and two groups (Control–Atherosclerosis). Values lower than cut-off indicates disease.

Comparison	AUC	Cutoff *	Sensitivity	Specificity	Accuracy
Control–Atorvastatin	0.734	12.5	1.000	0.396	0.561
Control–Rosuvastatin	0.780	10.8	1.000	0.542	0.676
Control–No statin	0.741	6.21	0.455	0.958	0.864
Control–Atherosclerosis	0.749	10.8	0.878	0.542	0.711

Note: * Mid-point of the plateau of thresholds that maximize the Youden index (Sensitivity + Specificity − 1); cut-off presented as a module. For each comparison, the rule is “classify as atherosclerosis when the index value ≤ cut-off”.

**Table 5:** Predicted parameters for sought function by LASSO approach.

Predictor	β Standardized	β Original Scale
Intercept	−0.147	5.254
Omega-6/3 Balance Index	−0.622	−0.145
C18:0/C18:1n-9	−1.416	−11.544
C20:4-n6/C22:4n-6	0.000	0.000

**Table 6:** Parameters of logistic diagnostic function optimized by LASSO approach, mean with standard deviation for groups, and results of *t*-test with Welch correction.

Atherosclerosis, Mean ± SD	Control, Mean ± SD	AUC	Brier Score	Cox-Snell R^2^	Nagelkerke R^2^	Calibration Slope
0.959 ± 0.849 *	−1.28 ± 1.65	0.880	0.137	0.423	0.564	1.122

Note: * Means here *p* < 0.0001.

**Table 7:** Results of ROC analysis and diagnostic performance of Youden (mid-plateau rule) cut-off for logistic diagnostic function and Cliff’s Δ for logit function. The comparison is between Atherosclerosis united groups and Control samples.

Cliff’s Δ	AUC (CI 95%)	Std. Error	*p*-Value	Cut-Off *	Sensitivity	Specificity	Accuracy
0.76 (−0.89−−0.62)	0.880 (0.811−0.941)	0.033	<0.0001	0.02	0.840	0.729	0.786

Note: * Mid-point of the plateau of thresholds that maximize the Youden index (Sensitivity + Specificity − 1); cut-off presented as a module. For each comparison, the rule is “classify as atherosclerosis when the index value ≤ cut-off”.

## Data Availability

The data can be provided at the official request of the Principal Investigator because our local ethics committee does not allow them to be provided openly.

## Supplementary Materials

The following supporting information can be downloaded at: https://www.mdpi.com/article/10.3390/biomedicines14010149/s1, Table S1. Inclusion and exclusion criteria; Table S2. Sample preparation and HPLC-MS/MS conditions; Table S3. Results of post hoc power analysis for 4 groups; Table S4. Cliff’s Δ (95% CI) calculated for comparison between Atherosclerosis united groups and Control samples; Table S5. ROC-curves’ parameters for single biomarkers in the plasma of the three studied groups with atherosclerosis in comparison to control; Table S6. Evaluation of reliability by Riley criteria of differences between statin-treated atherosclerosis groups and all united groups for LASSO modeling; Table S7. Diagnostic performance and three cut-off-Youden (mid-plateau rule), and maximum sensitivity and specificity for lognormal diagnostic function; Table S8. Cross-validated diagnostic performance and Youden cut-off stability for the logit model and O6/3-BI index for two groups (Control–Atherosclerosis).

## Abbreviations

The following abbreviations are used in this manuscript:

AA	Arachidonic Acid (C20:4n-6)
AA/EPA	Arachidonic-Acid-to-Eicosapentaenoic-Acid Ratio
AdA	Adrenic Acid (C22:4n-6)
AEA	Anandamide
AUC	Area Under the ROC Curve
AS	Atherosclerosis
CI	Confidence Interval
C18:0/C18:1n-9	Stearic-to-Oleic-Acid Ratio (SFA/MUFA ratio)
C20:4n-6/C22:4n-6	Arachidonic-to-Adrenic-Acid Ratio
CV	Cross-Validation
DHA	Docosahexaenoic Acid (C22:6n-3)
DHEA	Docosahexaenoyl Ethanolamide
DPA	Docosapentaenoic Acid (C22:5n-3)
DPA/DHA	DPA-to-DHA Ratio
ELOVL2/5	Elongation of Very Long-Chain Fatty Acids Enzymes
EPA	Eicosapentaenoic Acid (C20:5n-3)
EPA/DHA	EPA-to-DHA Ratio
EPA/DPA	EPA-to-DPA Ratio
EPEA	Eicosapentaenoyl Ethanolamide
FA	Fatty Acid
FADS1/FADS2	Fatty Acid Desaturase 1 and 2
FNs	False Negatives
FPs	False Positives
HMG-CoA	Hydroxymethylglutaryl-Coenzyme A
JELIS	Japan EPA Lipid Intervention Study
LASSO	Least Absolute Shrinkage and Selection Operator
LC-MS/MS	Liquid Chromatography–Tandem Mass Spectrometry
LDL-C	Low-Density Lipoprotein Cholesterol
Logit	Logistic Regression Function Value
MCC	Matthews Correlation Coefficient
MUFAs	Monounsaturated Fatty Acids
NIR	No-Information Rate
O6/3-BI	Omega-6/3 Balance Index
Omega-3 Index	Sum of EPA and DHA (%) in RBC membranes
Omega-3 Status	Sum of EPA and DHA (%) in blood plasma
Omega-6/3	Total Omega-6/Total Omega-3 Ratio
PCSK9	Proprotein Convertase Subtilisin/Kexin Type 9
PPAR-α	Peroxisome Proliferator-Activated Receptor Alpha
PRI	Polyunsaturated Remodeling Index
PUFAs	Polyunsaturated Fatty Acids
RBC	Red Blood Cells
ROC	Receiver Operating Characteristic
SCD1	Stearoyl-CoA Desaturase-1
SD	Standard Deviation
SFAs	Saturated Fatty Acids
SPM	Specialized Pro-Resolving Mediators
SREBP-1c	Sterol Regulatory Element-Binding Protein 1c
TNs	True Negatives
TPs	True Positives
TRIPOD	Transparent Reporting of A Multivariable Prediction Model for Individual Prognosis Or Diagnosis

## References

[1] Watanabe T., Fan J. (2025). Atherosclerosis Is a Global No. 1 Killer. Atherosclerosis.

[2] Ibanez B., Bundgaard H. (2025). REACT Initiative: Early Cure of Atherosclerosis through Precision Prevention. Eur. Heart J..

[3] Poznyak A.V., Sukhorukov V.N., Eremin I.I., Nadelyaeva I.I., Orekhov A.N. (2023). Diagnostics of Atherosclerosis: Overview of the Existing Methods. Front. Cardiovasc. Med..

[4] Bordeianu G., Mitu I., Stanescu R.S., Ciobanu C.P., Petrescu-Danila E., Marculescu A.D., Dimitriu D.C. (2022). Circulating Biomarkers for Laboratory Diagnostics of Atherosclerosis—Literature Review. Diagnostics.

[5] Yunoki K., Matsumi H., Miyoshi T., Kubo M., Hata Y., Yuasa S. (2025). Clinical Significance of Serum Omega-3 Fatty Acids on Endothelial Function in Patients with Coronary Artery Disease Under Statin Therapy. J. Cardiovasc. Dev. Dis..

[6] Alfaddagh A., Martin S.S., Leucker T.M., Michos E.D., Blaha M.J., Lowenstein C.J., Jones S.R., Toth P.P. (2020). Inflammation and Cardiovascular Disease: From Mechanisms to Therapeutics. Am. J. Prev. Cardiol..

[7] Kotlyarov S., Kotlyarova A. (2022). Involvement of Fatty Acids and Their Metabolites in the Development of Inflammation in Atherosclerosis. Int. J. Mol. Sci..

[8] Glaser C., Demmelmair H., Koletzko B. (2010). High-Throughput Analysis of Total Plasma Fatty Acid Composition with Direct In Situ Transesterification. PLoS ONE.

[9] Davinelli S., Intrieri M., Corbi G., Scapagnini G. (2021). Metabolic Indices of Polyunsaturated Fatty Acids: Current Evidence, Research Controversies, and Clinical Utility. Crit. Rev. Food Sci. Nutr..

[10] Menotti A., Puddu P.E. (2024). Dietary Fatty Acids Predicting Long Term Cardiovascular Mortality in a Cohort of Middle-Aged Men Followed-Up until Extinction. Hearts.

[11] Akbar S., Bhatti M.Z., Saeed R.F., Qazi A.S. (2021). Polyunsaturated Fatty Acids: Impact on Health and Disease Status. Life Sci..

[12] Djuricic I., Calder P.C. (2024). Omega-3 (n-3) Fatty Acid–Statin Interaction: Evidence for a Novel Therapeutic Strategy for Atherosclerotic Cardiovascular Disease. Nutrients.

[13] López-Vicario C., Rius B., Alcaraz-Quiles J., García-Alonso V., Lopategi A., Titos E., Clària J. (2016). Pro-Resolving Mediators Produced from EPA and DHA: Overview of the Pathways Involved and Their Mechanisms in Metabolic Syndrome and Related Liver Diseases. Eur. J. Pharmacol..

[14] Chen Y., Wang J., Nie R., Zhou S. (2008). Endogenous Pro-Resolving and Anti-Inflammatory Lipid Mediators: The New Hope of Atherosclerotic Diseases. Med. Hypotheses.

[15] Radbakhsh S., Katsiki N., Santos R.D., Mikhailidis D.P., Mantzoros C.S., Sahebkar A. (2022). Effects of Statins on Specialized Pro-Resolving Mediators: An Additional Pathway Leading to Resolution of Inflammation. Metabolism.

[16] DiNicolantonio J.J., O’Keefe J.H. (2018). Importance of Maintaining a Low Omega–6/Omega–3 Ratio for Reducing Inflammation. Open Heart.

[17] Simonetto M., Infante M., Sacco R.L., Rundek T., Della-Morte D. (2019). A Novel Anti-Inflammatory Role of Omega-3 PUFAs in Prevention and Treatment of Atherosclerosis and Vascular Cognitive Impairment and Dementia. Nutrients.

[18] Luo C., Chen Z. (2022). Is Omega-3 Index Necessary for Fish Oil Supplements for CVD Risk Prevention?. Cardiol. Plus.

[19] Harris W.S. (2025). Recent Studies Confirm the Utility of the Omega-3 Index. Curr. Opin. Clin. Nutr. Metab. Care.

[20] Oppedisano F., Macrì R., Gliozzi M., Musolino V., Carresi C., Maiuolo J., Bosco F., Nucera S., Caterina Zito M., Guarnieri L. (2020). The Anti-Inflammatory and Antioxidant Properties of n-3 PUFAs: Their Role in Cardiovascular Protection. Biomedicines.

[21] Itakura H., Yokoyama M., Matsuzaki M., Saito Y., Origasa H., Ishikawa Y., Oikawa S., Sasaki J., Hishida H., Kita T. (2011). Relationships between Plasma Fatty Acid Composition and Coronary Artery Disease. J. Atheroscler. Thromb..

[22] Ishihara N., Suzuki S., Tanaka S., Watanabe Y., Nagayama D., Saiki A., Tanaka T., Tatsuno I. (2017). Atorvastatin Increases Fads1, Fads2 and Elovl5 Gene Expression via the Geranylgeranyl Pyrophosphate-Dependent Rho Kinase Pathway in 3T3-L1 Cells. Mol. Med. Rep..

[23] Tutino V., De Nunzio V., Caruso M.G., Bonfiglio C., Franco I., Mirizzi A., De Leonardis G., Cozzolongo R., Giannuzzi V., Giannelli G. (2018). Aerobic Physical Activity and a Low Glycemic Diet Reduce the AA/EPA Ratio in Red Blood Cell Membranes of Patients with NAFLD. Nutrients.

[24] Gouaref I., Bouazza A., Abderrhmane S.A., Koceir E.-A. (2020). Lipid Profile Modulates Cardiometabolic Risk Biomarkers Including Hypertension in People with Type-2 Diabetes: A Focus on Unbalanced Ratio of Plasma Polyunsaturated/Saturated Fatty Acids. Molecules.

[25] Yokoyama M., Origasa H., Matsuzaki M., Matsuzawa Y., Saito Y., Ishikawa Y., Oikawa S., Sasaki J., Hishida H., Itakura H. (2007). Effects of Eicosapentaenoic Acid on Major Coronary Events in Hypercholesterolaemic Patients (JELIS): A Randomised Open-Label, Blinded Endpoint Analysis. Lancet.

[26] Lee Y., Lai H.T.M., de Oliveira Otto M.C., Lemaitre R.N., McKnight B., King I.B., Song X., Huggins G.S., Vest A.R., Siscovick D.S. (2020). Serial Biomarkers of De Novo Lipogenesis Fatty Acids and Incident Heart Failure in Older Adults: The Cardiovascular Health Study. J. Am. Hear. Assoc..

[27] Balatskyi V.V., Dobrzyn P. (2023). Role of Stearoyl-CoA Desaturase 1 in Cardiovascular Physiology. Int. J. Mol. Sci..

[28] Piccinin E., Cariello M., De Santis S., Ducheix S., Sabbà C., Ntambi J.M., Moschetta A. (2019). Role of Oleic Acid in the Gut-Liver Axis: From Diet to the Regulation of Its Synthesis via Stearoyl-CoA Desaturase 1 (SCD1). Nutrients.

[29] Lounis M.A., Bergeron K.-F., Burhans M.S., Ntambi J.M., Mounier C. (2017). Oleate Activates SREBP-1 Signaling Activity in SCD1-Deficient Hepatocytes. Am. J. Physiol.-Endocrinol. Metab..

[30] Matsui H., Yokoyama T., Sekiguchi K., Iijima D., Sunaga H., Maniwa M., Ueno M., Iso T., Arai M., Kurabayashi M. (2012). Stearoyl-CoA Desaturase-1 (SCD1) Augments Saturated Fatty Acid-Induced Lipid Accumulation and Inhibits Apoptosis in Cardiac Myocytes. PLoS ONE.

[31] Menotti A., Puddu P.E. (2025). Ancel Keys, the Mediterranean Diet, and the Seven Countries Study: A Review. J. Cardiovasc. Dev. Dis..

[32] Bock M., von Schacky C., Scherr J., Lorenz E., Lechner B., Krannich A., Wachter R., Duvinage A., Edelmann F., Lechner K. (2023). De Novo Lipogenesis-Related Monounsaturated Fatty Acids in the Blood Are Associated with Cardiovascular Risk Factors in HFpEF Patients. J. Clin. Med..

[33] Bhatt D.L., Steg P.G., Miller M., Brinton E.A., Jacobson T.A., Ketchum S.B., Doyle R.T., Juliano R.A., Jiao L., Granowitz C. (2019). Cardiovascular Risk Reduction with Icosapent Ethyl for Hypertriglyceridemia. N. Engl. J. Med..

[34] Alfaddagh A., Elajami T.K., Saleh M., Mohebali D., Bistrian B.R., Welty F.K. (2019). An Omega-3 Fatty Acid Plasma Index ≥4% Prevents Progression of Coronary Artery Plaque in Patients with Coronary Artery Disease on Statin Treatment. Atherosclerosis.

[35] Peña N., Amézaga J., Marrugat G., Landaluce A., Viar T., Arce J., Larruskain J., Lekue J., Ferreri C., Ordovás J.M. (2023). Competitive Season Effects on Polyunsaturated Fatty Acid Content in Erythrocyte Membranes of Female Football Players. J. Int. Soc. Sports Nutr..

[36] Wang Z., Gao H., Ma X., Zhu D., Zhao L., Xiao W. (2024). Adrenic Acid: A Promising Biomarker and Therapeutic Target (Review). Int. J. Mol. Med..

[37] Brouwers H., Jónasdóttir H.S., Kuipers M.E., Kwekkeboom J.C., Auger J.L., Gonzalez-Torres M., López-Vicario C., Clària J., Freysdottir J., Hardardottir I. (2020). Anti-Inflammatory and Proresolving Effects of the Omega-6 Polyunsaturated Fatty Acid Adrenic Acid. J. Immunol..

[38] Kim S.R., Jeon S.Y., Lee S.-M. (2015). The Association of Cardiovascular Risk Factors with Saturated Fatty Acids and Fatty Acid Desaturase Indices in Erythrocyte in Middle-Aged Korean Adults. Lipids Health Dis..

[39] Khaw K.T., Friesen M.D., Riboli E., Luben R., Wareham N. (2012). Plasma Phospholipid Fatty Acid Concentration and Incident Coronary Heart Disease in Men and Women: The EPIC-Norfolk Prospective Study. PLoS Med..

[40] Eroshchenko N., Danilova E., Lomonosova A., Antonik A., Lebedeva S., Gognieva D., Shchekochikhin D., Demura T., Krot M., Gogiberidze N. (2025). Plasma Fatty Acid Profiling and Mathematical Estimation of the Omega-3 Index: Toward Diagnostic Tools in Atherosclerosis and Statin Therapy Monitoring. Biomedicines.

[41] Tanaka S., Ishihara N., Suzuki S., Watanabe Y., Nagayama D., Yamaguchi T., Ohira M., Saiki A., Tanaka T., Tatsuno I. (2019). Fatty Acid Desaturase 2 Is Up-Regulated by the Treatment with Statin through Geranylgeranyl Pyrophosphate-Dependent Rho Kinase Pathway in HepG2 Cells. Sci. Rep..

[42] Eroshchenko N.N., Veselov V.V., Pirogov A.V., Danilova E.Y., Kirushin A.N., Paravyan A.L., Cravotto G. (2023). Development and Validation of a HPLC-MS/MS Method for the Analysis of Fatty Acids—In the Form of FAME Ammonium Adducts—In Human Whole Blood and Erythrocytes to Determine Omega-3 Index. J. Chromatogr. B.

[43] Collins G.S., Reitsma J.B., Altman D.G., Moons K. (2015). Transparent Reporting of a Multivariable Prediction Model for Individual Prognosis or Diagnosis (TRIPOD): The TRIPOD Statement. BMC Med..

[44] Wang B., Wu L., Chen J., Dong L., Chen C., Wen Z., Hu J., Fleming I., Wang D.W. (2021). Metabolism Pathways of Arachidonic Acids: Mechanisms and Potential Therapeutic Targets. Signal Transduct. Target. Ther..

[45] Katoh K., Katoh Y., Kubo A., Iida M., Ikeda Y., Iwata T., Nishio H., Sugawara M., Kato D., Suematsu M. (2023). Serum Free Fatty Acid Changes Caused by High Expression of Stearoyl-CoA Desaturase 1 in Tumor Tissues Are Early Diagnostic Markers for Ovarian Cancer. Cancer Res. Commun..

[46] Gray N., Lawler N., Zeng A., Ryan M., Bong S., Boughton B., Bizkarguenaga M., Bruzzone C., Embade N., Wist J. (2021). Diagnostic Potential of the Plasma Lipidome in Infectious Disease: Application to Acute SARS-CoV-2 Infection. Metabolites.

[47] Schmieta H.M., Greupner T., Schneider I., Wrobel S., Christa V., Kutzner L., Hahn A., Harris W.S., Schebb N.H., Schuchardt J.P. (2025). Plasma Levels of EPA and DHA after Ingestion of a Single Dose of EPA and DHA Ethyl Esters. Lipids.

[48] Serhan C.N., Levy B.D. (2018). Resolvins in Inflammation: Emergence of the pro-Resolving Superfamily of Mediators. J. Clin. Investig..

[49] Youden W.J. (1950). Index for Rating Diagnostic Tests. Cancer.

[50] Fluss R., Faraggi D., Reiser B. (2005). Estimation of the Youden Index and Its Associated Cutoff Point. Biom. J..

[51] Schisterman E.F., Perkins N. (2007). Confidence Intervals for the Youden Index and Corresponding Optimal Cut-Point. Commun. Stat. Simul. Comput..

[52] Mustonen A.M., Nieminen P. (2023). Dihomo-γ-Linolenic Acid (20:3n-6)—Metabolism, Derivatives, and Potential Significance in Chronic Inflammation. Int. J. Mol. Sci..

[53] Dennis E.A., Norris P.C. (2015). Eicosanoid Storm in Infection and Inflammation. Nat. Rev. Immunol..

[54] Nikolic Turnic T., Arsic A., Vucic V., Petrovic S., Ristic-Medic D., Zivkovic V., Srejovic I., Jeremic J., Radonjic T., Milosavljevic I. (2019). Hydroxymethylglutaryl Coenzyme a Reductase Inhibitors Differentially Modulate Plasma Fatty Acids in Rats With Diet-Induced-Hyperhomocysteinemia: Is ω-3 Fatty Acids Supplementation Necessary?. Front. Physiol..

[55] Schooneveldt Y.L., Giles C., Keating M.F., Mellett N.A., Jurrjens A.W., Paul S., Calkin A.C., Meikle P.J. (2021). The Impact of Simvastatin on Lipidomic Markers of Cardiovascular Risk in Human Liver Cells Is Secondary to the Modulation of Intracellular Cholesterol. Metabolites.

[56] Sliz E., Kettunen J., Holmes M.V., Williams C.O., Boachie C., Wang Q., Männikkö M., Sebert S., Walters R., Lin K. (2018). Metabolomic Consequences of Genetic Inhibition of PCSK9 Compared With Statin Treatment. Circulation.

[57] Serhan C.N. (2014). Pro-Resolving Lipid Mediators Are Leads for Resolution Physiology. Nature.

[58] Serhan C.N., Petasis N.A. (2011). Resolvins and Protectins in Inflammation Resolution. Chem. Rev..

[59] Jun J.E., Jeong I.-K., Yu J.M., Kim S.R., Lee I.K., Han K.-A., Choi S.H., Kim S.-K., Park H.K., Mok J.-O. (2020). Efficacy and Safety of Omega-3 Fatty Acids in Patients Treated with Statins for Residual Hypertriglyceridemia: A Randomized, Double-Blind, Placebo-Controlled Clinical Trial. Diabetes Metab. J..

[60] Ridker P.M. (2016). Residual Inflammatory Risk: Addressing the Obverse Side of the Atherosclerosis Prevention Coin. Eur. Heart J..

[61] Boden W.E., Bhatt D.L., Toth P.P., Ray K.K., Chapman M.J., Lüscher T.F. (2020). Profound Reductions in First and Total Cardiovascular Events with Icosapent Ethyl in the REDUCE-IT Trial: Why These Results Usher in a New Era in Dyslipidaemia Therapeutics. Eur. Heart J..

[62] Hoang T., Kim J. (2020). Comparative Effect of Statins and Omega-3 Supplementation on Cardiovascular Events: Meta-Analysis and Network Meta-Analysis of 63 Randomized Controlled Trials Including 264,516 Participants. Nutrients.

[63] Bird J.K., Calder P.C., Eggersdorfer M. (2018). The Role of N-3 Long Chain Polyunsaturated Fatty Acids in Cardiovascular Disease Prevention, and Interactions with Statins. Nutrients.

[64] Berne C., Siewert-Delle A. (2005). Comparison of Rosuvastatin and Atorvastatin for Lipid Lowering in Patients with Type 2 Diabetes Mellitus: Results from the URANUS Study. Cardiovasc. Diabetol..

[65] Nozue T., Yamamoto S., Tohyama S., Fukui K., Umezawa S., Onishi Y., Kunishima T., Sato A., Nozato T., Miyake S. (2013). Effects of Statins on Serum N-3 to n-6 Polyunsaturated Fatty Acid Ratios in Patients With Coronary Artery Disease. J. Cardiovasc. Pharmacol. Ther..

[66] Gregory M.K., Cleland L.G., James M.J. (2013). Molecular Basis for Differential Elongation of Omega-3 Docosapentaenoic Acid by the Rat Elovl5 and Elovl2. J. Lipid Res..

[67] Moro K., Nagahashi M., Ramanathan R., Takabe K., Wakai T. (2016). Resolvins and Omega Three Polyunsaturated Fatty Acids: Clinical Implications in Inflammatory Diseases and Cancer. World J. Clin. Cases.

[68] Sprecher H. (2000). Metabolism of Highly Unsaturated N-3 and n-6 Fatty Acids. Biochim. Biophys. Acta (BBA)—Mol. Cell Biol. Lipids.

[69] Sahebkar A., Simental-Mendía L.E., Pedone C., Ferretti G., Nachtigal P., Bo S., Derosa G., Maffioli P., Watts G.F. (2016). Statin Therapy and Plasma Free Fatty Acids: A Systematic Review and Meta-analysis of Controlled Clinical Trials. Br. J. Clin. Pharmacol..

[70] Sparkes C., Sinclair A.J., Gibson R.A., Else P.L., Meyer B.J. (2020). High Variability in Erythrocyte, Plasma and Whole Blood EPA and DHA Levels in Response to Supplementation. Nutrients.

[71] Metherel A.H., Bazinet R.P. (2019). Updates to the N-3 Polyunsaturated Fatty Acid Biosynthesis Pathway: DHA Synthesis Rates, Tetracosahexaenoic Acid and (Minimal) Retroconversion. Prog. Lipid Res..

[72] Schulze M.B., Minihane A.M., Saleh R.N.M., Risérus U. (2020). Intake and Metabolism of Omega-3 and Omega-6 Polyunsaturated Fatty Acids: Nutritional Implications for Cardiometabolic Diseases. Lancet Diabetes Endocrinol..

[73] Basil M.C., Levy B.D. (2016). Specialized Pro-Resolving Mediators: Endogenous Regulators of Infection and Inflammation. Nat. Rev. Immunol..

[74] Chiang N., Serhan C.N. (2020). Specialized Pro-Resolving Mediator Network: An Update on Production and Actions. Essays Biochem..

[75] Gabbs M., Leng S., Devassy J.G., Monirujjaman M., Aukema H.M. (2015). Advances in Our Understanding of Oxylipins Derived from Dietary PUFAs. Adv. Nutr..

[76] Jannas-Vela S., Espinosa A., Candia A.A., Flores-Opazo M., Peñailillo L., Valenzuela R. (2023). The Role of Omega-3 Polyunsaturated Fatty Acids and Their Lipid Mediators on Skeletal Muscle Regeneration: A Narrative Review. Nutrients.

[77] Simopoulos A.P. (2020). Omega-6 and Omega-3 Fatty Acids: Endocannabinoids, Genetics and Obesity. OCL.

[78] Chilton F.H., Manichaikul A., Yang C., O’Connor T.D., Johnstone L.M., Blomquist S., Schembre S.M., Sergeant S., Zec M., Tsai M.Y. (2022). Interpreting Clinical Trials With Omega-3 Supplements in the Context of Ancestry and FADS Genetic Variation. Front. Nutr..

[79] Lee D.K. (2020). Data Transformation: A Focus on the Interpretation. Korean J. Anesthesiol..

[80] Hübner U., Englisch C., Werkmann H., Butz H., Georg T., Zabransky S., Herrmann W. (2002). Continuous Age-Dependent Reference Ranges for Thyroid Hormones in Neonates, Infants, Children and Adolescents Established Using the ADVIA(r) Centaur(Tm) Analyzer. Clin. Chem. Lab. Med..

[81] Yuan Z., Ghosh D. (2008). Combining Multiple Biomarker Models in Logistic Regression. Biometrics.

[82] Oboh A., Kabeya N., Carmona-Antoñanzas G., Castro L.F.C., Dick J.R., Tocher D.R., Monroig O. (2017). Two Alternative Pathways for Docosahexaenoic Acid (DHA, 22:6n-3) Biosynthesis Are Widespread among Teleost Fish. Sci. Rep..

[83] Voss A., Reinhart M., Sankarappa S., Sprecher H. (1991). The Metabolism of 7,10,13,16,19-Docosapentaenoic Acid to 4,7,10,13,16,19-Docosahexaenoic Acid in Rat Liver Is Independent of a 4-Desaturase. J. Biol. Chem..

[84] Cormier H., Rudkowska I., Lemieux S., Couture P., Julien P., Vohl M.-C. (2014). Effects of FADS and ELOVL Polymorphisms on Indexes of Desaturase and Elongase Activities: Results from a Pre-Post Fish Oil Supplementation. Genes Nutr..

[85] Kremmyda L.-S., Tvrzicka E., Stankova B., Zak A. (2011). Fatty Acids as Biocompounds: Their Role in Human Metabolism, Health and Disease—A Review. Part 2: Fatty Acid Physiological Roles and Applications in Human Health and Disease. Biomed. Pap..

[86] Sergeant S., Keith B.A., Seeds M.C., Legins J.A., Young C.B., Vitolins M.Z., Chilton F.H. (2023). Impact of FADS Gene Variation and Dietary Fatty Acid Exposure on Biochemical and Anthropomorphic Phenotypes in a Hispanic/Latino Cohort. Front. Nutr..

[87] Harris W.S., Luo J., Pottala J.V., Margolis K.L., Espeland M.A., Robinson J.G. (2016). Red Blood Cell Fatty Acids and Incident Diabetes Mellitus in the Women’s Health Initiative Memory Study. PLoS ONE.

[88] Matthan N.R., Ooi E.M., Van Horn L., Neuhouser M.L., Woodman R., Lichtenstein A.H. (2014). Plasma Phospholipid Fatty Acid Biomarkers of Dietary Fat Quality and Endogenous Metabolism Predict Coronary Heart Disease Risk: A Nested Case-Control Study Within the Women’s Health Initiative Observational Study. J. Am. Hear. Assoc..

[89] Jauregibeitia I., Portune K., Rica I., Tueros I., Velasco O., Grau G., Castaño L., Di Nolfo F., Ferreri C., Arranz S. (2021). Potential of Erythrocyte Membrane Lipid Profile as a Novel Inflammatory Biomarker to Distinguish Metabolically Healthy Obesity in Children. J. Pers. Med..

[90] Li C., Zhang W., Chen Q., Xiao F., Yang X., Xiao B., Cheng Y., Qin J., Li X., Wan D. (2025). Development and Validation of a Logistic Regression Model for the Diagnosis of Colorectal Cancer. Sci. Rep..

[91] Riley R.D., Ensor J., Snell K.I.E., Harrell F.E., Martin G.P., Reitsma J.B., Moons K.G.M., Collins G., Van Smeden M. (2020). Calculating the Sample Size Required for Developing a Clinical Prediction Model. BMJ.

[92] Tibshirani R. (1996). Regression Shrinkage and Selection Via the Lasso. J. R. Stat. Soc. Series B Stat. Methodol..

[93] DeLong E.R., DeLong D.M., Clarke-Pearson D.L. (1988). Comparing the Areas under Two or More Correlated Receiver Operating Characteristic Curves: A Nonparametric Approach. Biometrics.

[94] Van Calster B., McLernon D.J., van Smeden M., Wynants L., Steyerberg E.W. (2019). Calibration: The Achilles Heel of Predictive Analytics. BMC Med..

