# Supplementary material for: Comparative Diagnostic Performance of Conventional and Novel Fatty Acid Indices in Blood Plasma as Biomarkers of Atherosclerosis Under Statin Therapy
Source: Biomedicines. 2026 Jan 11;14(1):149. doi: 10.3390/biomedicines14010149 (PMC12839158; doi:10.3390/biomedicines14010149)
Supplement: Supplementary file 1 [file biomedicines-14-00149-s001.zip › biomedicines-4028072-supplementary.pdf]

Supplementary Material to the Article

Comparative Diagnostic Performance of Conventional and Novel Fatty Acid Indices in Blood Plasma as Biomarkers of Atherosclerosis Under Statin Therapy

Supplemental Information

Table S1. Inclusion and exclusion criteria.

Domain	Atherosclerosis group (AS)	Control group
**Inclusion criteria**	(1) Age 18–85 years; (2) written informed consent; (3) hemodynamically significant stenosing atherosclerosis of brachiocephalic arteries (BCA) confirmed by duplex ultrasound and/or MSCT angiography (with or without prior TIA/stroke attributed to carotid disease); (4) general blood analysis and lipid profile available; (5) outpatient follow-up or inpatient care at the research institution; and (6) undergoing carotid endarterectomy.	(1) Age 18–85 years; (2) written informed consent; (3) no clinical symptoms suggestive of atherosclerosis; (4) no detectable BCA atherosclerosis by duplex ultrasound and/or MSCT angiography; and (5) outpatient follow-up or inpatient care at the research institution.
**Non-inclusion / exclusion criteria**	(1) CKD stage ≥ 3b (eGFR <45 mL/min/1.73 m²); (2) severe non-cardiovascular somatic disease with life expectancy <6 months; (3) active exacerbation of chronic disease; (4) body weight <40 kg or >125 kg; and (5) pregnancy.	(1) History/clinical evidence of systemic atherosclerosis or cardiovascular disease; (2) autoimmune disease, malignancy, COPD, CKD stage ≥ 3, or liver cirrhosis; (3) acute infectious/inflammatory condition at sampling; (4) exacerbation of chronic illness; (5) current lipid-lowering, anti-inflammatory, or immunosuppressive therapy; (6) body weight <40 kg or > 125 kg; and (7) pregnancy.
**Allowed conditions (controls)**	—	Non-exacerbated diabetes mellitus; chronic gastritis; prior tonsillitis; seasonal allergic rhinitis; and well-controlled arterial hypertension (stage 1, without cardiovascular complications/target organ damage) were not exclusionary.
**Study-wide exclusion rule**	Withdrawal of informed consent at any stage.	Withdrawal of informed consent at any stage.

Abbreviations: AS, atherosclerosis; BCA, brachiocephalic arteries; CKD, chronic kidney disease; COPD, chronic obstructive pulmonary disease; eGFR, estimated glomerular filtration rate; MSCT, multislice computed tomography; TIA, transient ischemic attack.

Table S2. Sample preparation and HPLC-MS/MS conditions.

Stage	Key parameters (abbreviated)
**Sample preparation**	40 µL blood plasma (K3EDTA) + 50 µL IS (methyl heptadecanoate, C17:0 FAME; 500 µg/mL in MeOH)
	Extract with 910 µL MeOH with BHT 200 µg/m; shake 30 min at 2000 rpm, and centrifuge for 15 minutes (5000 g) at 4 °C
	Transfer 500 µL supernatant into a vial, methylation with 500 µL BF_3 15% in MeOH; 100 °C, 90 min
	Cool at 4 °C, and transfer 500 µL supernatant into a vial, mix with 500 µL deionized water.
**HPLC**	LC-20AD Prominence; Kinetex C8 (3.0×100 mm, 2.6 µm, 100 Å)
	Mobile phase A: deionized water + 0.1% formic acid + 5 mM ammonium formate
	Mobile phase A: ACN:IPA (1:1) 99% with 1% deionized water + 0.1% formic acid + 5 mM ammonium formate; gradient 20 min
	Flow 500 µL/min; column 40 °C; post-column split 100–170 µL/min into source, injection volume 2 µL
**MS/MS**	Sciex 4500 QTRAP, ESI(+), Scheduled MRM; ions $[M+NH4]^+$
	Source: CUR 35; CAD Medium; IS 5500 V; TEM 0; GS1 50; GS2 50
	List of MRM (Q1/Q3): C12:0 (232.3/215.3); C14:0 (260.3/243.3), C16:0 (288.3/271.3), C18:0 (316.2/299.2), C20:0 (344.3/327.3), C22:0 (372.3/355.3), C24:0 (400.3/383.3), C17:0 IS (302.3/285.3), C16:1 cis (286.3/269.3), C18:1 cis (314.3/297.3), C20:1 cis (342.3/325.3), C24:1 cis (398.3/381.3), C16:1 trans (286.3/269.3), C18:1 trans (314.3/297.3), C18:2-trans (312.3/295.3), C18:2 n-6 (312.3/295.3), C18:3 n-6 (310.3/293.3), C20:2 n-6 (340.3/323.3), C20:3 n-6 (338.3/321.3), C20:4 n-6 (336.3/319.3), C22:4 n-6 (364.3/347.3), DPA C22:5n-3 (362.3/345.3), EPA C20:5n-3 (334.3/317.3), and DHA C22:6n-3 (360.3/343.3)
**Quantification**	Analyst 1.6.3 / MultiQuant 3.0; peak area ratio (analyte/IS); weighted ($1/x^2$) linear regression; r ≥0.99; ≥6 points of calibration levels
**Calibration levels**	Analyte group 1 (C12:0, C16:0, and C18:0): 250 – 8000 ng/ml
	Analyte group 2 (C20:0, C22:0, C24:0; C16:1 cis, C20:1 cis, C24:1 cis; C16:1 trans, C18:1 trans, and C18:2 trans): 62.5 – 2000 ng/ml
	Analyte group 3 (C14:0; C18:1 cis; C18:2 n-6; C18:3 n-6; C20:2 n-6; C20:3 n-6; C20:4 n-6; C22:4 n-6; DPA C22:5 n-3; EPA C20:5 n-3; and DHA C22:6 n-3): 62.5 – 8000 ng/ml

Abbreviations: ACN, acetonitrile; BF_3, boron trifluoride; CAD, collision gas; CUR, curtain gas; ESI(+), positive electrospray ionization; FAME, fatty acid methyl ester; GS1/GS2, ion source gases; IPA, isopropanol; IS, internal standard; MeOH, methanol; MRM, multiple reaction monitoring; QC, quality control.

Table S3. Results of post hoc power analysis for 4 groups.

Parameter	90% power, α=0.05	
	**Cohen's f**	**Sample size per group**
C18:0	3.78	40
C18:1 n-9	4.25	40
C20:3n-6	1.18	15
C20:4 n-6 (AA)	1.80	10
C22:4 n-6 (AdA)	0.80	27
DPA	1.33	13
EPA	1.42	12
DHA	1.13	16

Table S4. Cliff's Δ (95 % CI) calculated for ccomparison between Atherosclerosis united groups and Control samples.

Parameters	Cliff's Δ (95 % CI)
C18:0	0.59 (0.40–0.75)
C18:1n-9	–0.68 (-0.83– –0.50)
C20:3n-6	0.22 (–0.02–0.43)
C20:4n-6	0.32 (0.08–0.55)
C22:4n-6	–0.17 (–0.39–0.07)
DPA, C22:5 n-3	0.22 (–0.02–0.44)
EPA, C20:5 n-3	0.28 (0.05–0.49)
DHA, C22:6 n-3	0.23 (–0.02–0.46)
Omega-3 Status	0.29 (0.06–0.52)
AA/EPA	–0.20 (–0.41–0.02)
Omega 6/3	–0.25 (–0.48—0.02)
C20:4-n6/C22:4n-6	0.41 (0.19–0.61)
C18:0/C18:1n-9	0.72 (0.56–0.84)
Omega-6/3 Balance Index	0.49 (0.29–0.67)

Table S5. ROC-curves' parameters for single biomarkers in the plasma of the three studied groups with atherosclerosis in comparison to control.

Parameters	Control–Atorvastatin				Control–Rosuvasatin				Control–No statin			
	AUC	Std. Error	95% CI	P-value	AUC	Std. Error	95% CI	P-value	AUC	Std. Error	95% CI	P-value
**Selected FA: SFA, MUFA, omega-6, -3 pathways**												
C18:0	0.785	0.054	0.678 – 0.891	0.0003	0.793	0.053	0.689 – 0.898	<0.0001	0.819	0.064	0.693 – 0.945	0.0010
C18:1n-9	0.827	0.051	0.728 – 0.927	<0.0001	0.863	0.046	0.773 – 0.953	<0.0001	0.856	0.054	0.751 – 0.962	0.0002
C20:3n-6	0.609	0.084	0.444 – 0.773	0.1685	0.640	0.073	0.498 – 0.782	0.0659	0.587	0.101	0.389 – 0.785	0.3706
C20:4n-6	0.671	0.067	0.538 – 0.802	0.0295	0.711	0.068	0.577 – 0.844	0.0054	0.637	0.089	0.462 – 0.812	0.1567
C22:4n-6	0.576	0.076	0.428 – 0.725	0.3301	0.503	0.069	0.368 – 0.639	0.9648	0.601	0.079	0.447 – 0.755	0.2978
DPA, C22:5 n-3	0.512	0.080	0.354 – 0.669	0.8825	0.615	0.067	0.483 – 0.747	0.1274	0.671	0.076	0.522 – 0.819	0.0778
EPA, C20:5 n-3	0.591	0.076	0.442 – 0.740	0.2517	0.628	0.072	0.487 – 0.768	0.1021	0.682	0.085	0.518 – 0.846	0.0634
DHA, C22:6 n-3	0.573	0.069	0.436 – 0.710	0.3505	0.612	0.065	0.484 – 0.739	0.1472	0.647	0.069	0.513 – 0.782	0.1286

Table S6. Evaluation of reliability by Riley criteria of differences between statin-treated atherosclerosis groups and all united groups for LASSO modeling.

Comparison	Marker	n	Events	Prev	Shrinkage S†	Nagelkerke R^2	n_{req} (Δ = 0.10)	Meets all Riley criteria
Control–Atorvastatin	Omega-3 Status	69	19	0.28	0.67	0.04	78	No
	AA/EPA	69	19	0.28	0.37	0.01	78	No
	Omega-6/3	69	19	0.28	0	0	78	No
	C18:0/C18:1n-9	69	19	0.28	0.96	0.43	78	No
	C20:4-n6/C22:4n-6	69	19	0.28	0.91	0.2	78	No
	O6/3-BI	69	19	0.28	0.92	0.23	78	No
Control–Rosuvastatin	Omega-3 Status	71	21	0.29	0.83	0.10	80	No
	AA/EPA	71	21	0.29	0.56	0.03	80	No
	Omega-6/3	71	21	0.29	0.05	0.01	80	No
	C18:0/C18:1n-9	71	21	0.29	0.96	0.46	80	No
	C20:4-n6/C22:4n-6	71	21	0.29	0.89	0.16	80	No
	O6/3-BI	71	21	0.29	0.94	0.31	80	No
Control–No statin	Omega-3 Status	62	12	0.19	0.83	0.13	58	No
	AA/EPA	62	12	0.19	0.80	0.11	58	No
	Omega-6/3	62	12	0.19	0.34	0.01	58	No
	C18:0/C18:1n-9	62	12	0.19	0.94	0.37	58	Yes
	C20:4-n6/C22:4n-6	62	12	0.19	0.81	0.11	58	No
	O6/3-BI	62	12	0.19	0.89	0.2	58	No
Control–Atherosclerosis	Omega-3 Status	102	52	0.51	0.89	0.11	96	No
	AA/EPA	102	52	0.51	0.76	0.04	96	No
	Omega-6/3	102	52	0.51	0.17	0.01	96	No
	C18:0/C18:1n-9	102	52	0.51	0.98	0.48	96	Yes
	C20:4-n6/C22:4n-6	102	52	0.51	0.94	0.21	96	Yes
	O6/3-BI	102	52	0.51	0.96	0.30	96	Yes

Note: According to the last column, generally, the statin-stratified comparisons (Control vs atorvastatin/rosuvastatin/no statin) are underpowered (noted as No) for stable multivariable model development by Riley criteria (i.e., the required sample size exceeds the available n and expected shrinkage is insufficient). In contrast, most of the pooled groups' comparison (Control vs all atherosclerosis) meets feasibility thresholds, supporting development of a single pooled, statin-resistant model.

Table S7. Diagnostic performance and three cut-off-Youden (mid-plateau rule), and maximum sensitivity and specificity for lognormal diagnostic function.

Parameter	Comparison	Cohen's κ	Confusion Matrix	PPV	NPV	p (Acc<NIR)	Mc Nemar
O6/3-BI	Control–Atorvastatin	0.263	TN=19, FP=29, FN=0, TP=18	0.383	1.000	0.999	2.00e-07
	Control–Rosuvastatin	0.410	TN=26, FP=22, FN=0, TP=20	0.476	1.000	0.750	7.56e-06
	Control–No statin	0.480	TN=46, FP=2, FN=6, TP=5	0.714	0.885	0.205	0.289
	Control–Atherosclerosis	0.421	TN=26, FP=22, FN=6, TP=43	0.662	0.812	2.92e-05	0.005
Logit function	Control–Atherosclerosis	0.570	TN=35, FP=13, FN=8, TP=42	0.764	0.814	1.70e-08	3.83e-01

Table S8. Cross-validated diagnostic performance and Youden cut-off stability for the logit model and O6/3-BI index for two groups (Control–Atherosclerosis).

Score, CI 95%	O6/3-BI	Logit function
AUC	0.246 [0.161, 0.353]	0.880 [0.811, 0.941]
Sensitivity	0.976 [0.927, 1.000]	0.894 [0.805, 0.962]
Specificity	0.0 [0.000, 0.000]	0.717 [0.588, 0.837]
Accuracy	0.493 [0.398, 0.596]	0.806 [0.728, 0.880]
Balanced Accuracy	0.488 [0.463, 0.500]	0.805 [0.727, 0.876]
MCC	–0.111 [–0.197, –0.041]	0.621 [0.470, 0.763]
LR plus	0.976 [0.927, 1.000]	3.155 [2.140, 5.551]
LR minus	∞	0.148 [0.049, 0.277]
PPV	0.499 [0.402, 0.608]	0.763 [0.646, 0.864]
NPV	0.0 [0.000, 0.000]	0.869 [0.750, 0.951]
Median threshold	3.94	0.02
IQR low	3.94	0.02
IQR high	3.94	0.02
Min threshold	3.94	–0.146
Max threshold	4.84	0.124
Std threshold	0.161	0.036
